# A Universal Electrochemical Synthetic Strategy for the Direct Assembly of Single‐Atom Catalysts

**DOI:** 10.1002/advs.202304656

**Published:** 2023-10-12

**Authors:** Ya‐Kun Lv, Kun Wang, Wen‐Yan Sun, Peng Peng, Shuang‐Quan Zang

**Affiliations:** ^1^ Henan Key Laboratory of Crystalline Molecular Functional Materials Henan International Joint Laboratory of Tumor Theranostical Cluster Materials Green Catalysis Center and College of Chemistry Zhengzhou University Zhengzhou 450001 China

**Keywords:** electrocatalysis, electrochemical synthesis, hydrogen evolution reaction, metal–organic frameworks, single‐atom catalysts

## Abstract

Single‐atom catalysts (SACs) have been one of the frontiers in the field of catalysis in recent years owing to their high atomic utilization and unique electronic structure. To facilitate the practical application of single‐atom, it is vital to develop a sustainable, facile single‐atom preparation method with mass production potential. Herein, a universal one‐step electrochemical synthesis strategy is proposed, and various metal–organic framework‐supported SACs (including Pt, Au, Ir, Pd, Ru, Mo, Rh, and W) are straightforwardly obtained by simply replacing the guest metal precursors. As a proof‐of‐concept, the electrosynthetic Pt‐based catalysts exhibit outstanding activity and stability in the electrocatalytic hydrogen evolution reaction (HER). This study not only enriches the single‐atom synthesis methodology, but also extends the scenario of electrochemical synthesis, opening up new avenues for the design of advanced electro‐synthesized catalysts.

## Graphical abstract

A sustainable and universal one‐step electrochemical synthesis strategy is used to prepare catalysts with various single atomic sites such as Pt, Au, Ir, Pd, Ru, Mo, Rh, and W, among which the as‐synthetic Pt‐based catalysts exhibit outstanding activity and stability in the electrocatalytic hydrogen evolution reaction, extending the implications of electrochemical synthesis and the design of advanced electro‐synthesized catalysts.

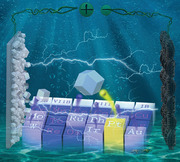

## 1 Introduction

Single‐atom catalysts (SACs) realize the anchoring of metallic components as individual atoms on solid supports.^[^ ^1^, ^2^, ^3^, ^4^ ^]^ As the dispersion of metal reaches a single atom state, the ultimate atom utilization, tunable coordination environment and interaction with carriers endow SACs with a wide application potential.^[^ ^5^, ^6^, ^7^, ^8^, ^9^ ^]^ By far, SACs are among the most popular materials especially in the field of catalysis, showing excellent catalytic performance such as CO_2_ reduction, water splitting, oxygen reduction reaction, and methane conversion.^[^ ^10^, ^11^, ^12^, ^13^, ^14^, ^15^, ^16^ ^]^ Nevertheless, the development of universal synthesis methods with mass production potential is still an obstacle to the practical application of SACs.^[^ ^3^, ^17^, ^18^, ^19^, ^20^ ^]^

Currently, a large number of attempts have been made to construct SACs, such as coprecipitation, atomic layer deposition, photo deposition, chemical vapor deposition, mechanochemical abrasion, and thermal shockwave.^[^ ^21^, ^22^, ^23^, ^24^, ^25^, ^26^ ^]^ These strategies often require complex synthesis processes (for example, complex single atom precursor preparation process or defect construction process) or specific equipment with harsh physiochemical conditions, which inevitably increase the total cost of SACs.^[^ ^27^, ^28^, ^29^, ^30^ ^]^ In this context, to explore the potential capabilities and widen the practical applications of SACs, it is crucial and has attracted numerous attention all over the world to develop universal, reproducible, and low‐cost routes for scalable preparation of SACs.^[^ ^17^, ^20^, ^31^ ^]^

Recently, electrochemical synthesis has been developed as a sustainable and atom‐economical strategy for the construction of challenging chemical products.^[^ ^32^, ^33^, ^34^, ^35^, ^36^, ^37^ ^]^ With electrons as traceless redox reagents, electrochemical synthesis could be precisely controlled by adjusting the applied conditions such as potential and electrolysis time.^[^ ^34^, ^36^ ^]^ Additionally, this ambient electrosynthesis strategy allows for the production of target products at scale, requiring only inexpensive potentiostat.^[^ ^34^, ^36^, ^38^ ^]^ However, despite electrochemical synthesis techniques that have been explored extensively, including the preparation of organic molecules, metal–organic frameworks, and inorganic nanomaterials, their application in the production of SACs remains to be understood in depth.^[^ ^34^, ^35^, ^38^, ^39^, ^40^, ^41^ ^]^

Herein, we develop a one‐step electrochemical aspproach to construct zeolitic imidazolate frameworks‐8 (ZIF‐8) with assembled single metal atoms, offering a novel and scalable strategy to synthesize SACs. Atomically dispersed metal atoms were assembled in ZIF‐8 (eZIF‐M) via the dynamic electrochemical process. Especially, by simply varying the metal sources, eZIF‐M with various single metal atoms (M stands for Pt, Au, Ir, Pd, Ru, Mo, Rh, and W) have been obtained, confirming the universality of this strategy. Furthermore, as a proof‐of‐concept, pyrolyzed eZIF‐Pt was evaluated by electrocatalytic HER, displaying promising potential applications as electrocatalysts.

## 2 Results and Discussion

### 2.1 Synthesis and Structural Characterization of eZIF‐M

**Figure 1:**
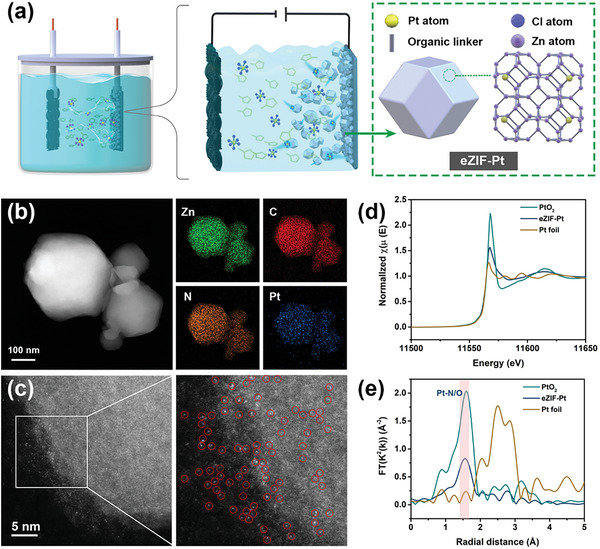
Preparation and characterizations of eZIF‐Pt. a) Schematic illustrating the synthesis of eZIF‐Pt. b) EDS element mapping and c) AC HAADF‐STEM images of eZIF‐Pt. d) Normalized XANES spectra and e) k^2^‐weighted FT spectra from EXAFS at the Pt L_3_‐edge of eZIF‐Pt, PtO_2_, and Pt foil.

The ZIF‐8 involving single‐metal atoms was synthesized by a one‐step dynamic electrosynthesis strategy. Taking the electrochemical synthesis of eZIF‐Pt as an example, we used the Zn sheet as the working electrode and K_2_PtCl_6_ as the metal precursor. Under cyclic potential scanning, the Zn sheet can slowly release Zn^2+^ and coordinate with 2‐methylimidazole in the electrolyte to form ZIF‐8, while the Pt species in the electrolyte were assembled in situ in ZIF‐8 (**Figure** **1**a). The assembly mechanism was attributed to Pt species replacing some of the Zn^2+^ in ZIF‐8 due to the Hard‐Soft Acid‐Base principle. Powder X‐ray diffraction (PXRD) and scanning electron microscope (SEM) results indicated that polyhedra ZIF‐8 with different sizes were synthesized by the dynamic electrochemical method (Figures S1 and S2, Supporting Information). The overall size distribution of eZIF‐Pt was estimated by dynamic light scattering and most crystals were found to be in the range of 10–200 nm (Figure S3, Supporting Information). Transmission electron microscopy (TEM) and high‐angle annular dark‐field scanning TEM (HAADF‐STEM) associated with Energy‐dispersive spectroscopic (EDS) elemental mapping experiments showed that Pt species were uniformly assembled in ZIF‐8 without obvious agglomeration (Figure 1b; Figure S4, Supporting Information). Further, from the aberration‐corrected (AC) HAADF‐STEM images of eZIF‐Pt, Pt single atoms were clearly observed as bright dots labeled with red circles (Figure 1c).

The electronic structure and coordination environment of eZIF‐Pt were further discussed by X‐ray absorption spectroscopy (XAS) and X‐ray photoelectron spectroscopy (XPS). As shown by normalized X‐ray absorption near‐edge spectroscopy (XANES) in Figure 1d, the white line peak intensity of eZIF‐Pt was between Pt foil and PtO_2_, indicating the presence of Pt^δ+^ species (0<δ<4) in eZIF‐Pt.^[^ ^42^ ^]^ Moreover, the adsorption threshold energies (E_0_) of Pt species obtained from the first derivative in PtO_2_, eZIF‐Pt, and Pt foil were 11 566.5, 11 565.1, and 11 563.7 eV, respectively (Figure S5, Supporting Information). The average oxidation state of Pt in eZIF‐Pt was thus calculated to be +2, which was consistent with the results obtained by XPS (Figure S6, Supporting Information). The decrease in the average oxidation number indicated that Pt was partially reduced during the electrochemical synthesis. To further study the coordination environment of eZIF‐Pt, we evaluated the local atomic structure of single‐atom Pt by extended X‐ray absorption fine structure (EXAFS). Fourier transforms EXAFS spectrum of eZIF‐Pt showed one main peak at 1.68 Å, corresponding to the peak of the Pt─N bond (Figure 1e). A Pt−Pt peak at ≈2.64 Å for Pt foil was not observed in eZIF‐Pt, confirming the sole existence of Pt single atoms. Moreover, the quantitative EXAFS fitting of the eZIF‐Pt was analyzed to obtain the local structural parameters (Figures S7 and S8, Supporting Information). The structural parameters of the eZIF‐Pt are exhibited in Table S1 (Supporting Information). Based on the fitting, the Pt−N coordination number was 2, indicating that Pt species were coordinated to N in 2‐methylimidazole during the electrochemical reaction. The Pt−N coordination environment in eZIF‐Pt was further verified by wavelet transform (WT) of its Pt L_3_‐edge, where a maximum peak intensity (3.75 Å^−1^) quite different from that of the Pt foil was observed (Figure S9, Supporting Information). This was further supported by Fourier‐transform infrared spectroscopy measurements, which confirm that in addition to the 427 cm^−1^ Zn−N band, an additional band of 554 cm^−1^ attributable to Pt−N can be observed in eZIF‐Pt (Figure S10, Supporting Information).^[^ ^43^, ^44^ ^]^

**Figure 2:**
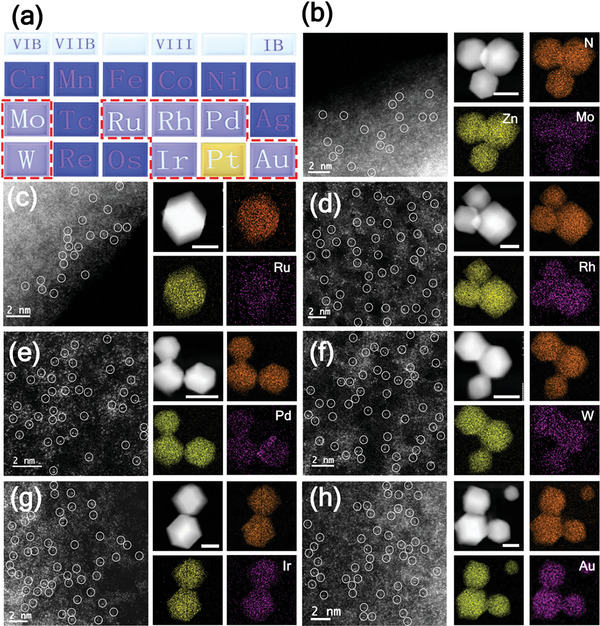
Characterization of eZIF‐M. a) Elements that are recommended for dynamic electrosynthesis strategy. AC HAADF STEM, HAADF STEM, and corresponding EDS element mapping images of b) eZIF‐Mo, c) eZIF‐Ru, d) eZIF‐Rh, e) eZIF‐Pd, f) eZIF‐W, g) eZIF‐Ir, and h) eZIF‐Au. The length of the scale bars in HAADF STEM is 100 nm.

Dynamic electrochemical synthesis strategy can be generically used to prepare a series of metal SACs. By simply replacing K_2_PtCl_6_ with other metal precursors, including HAuCl_4_·xH_2_O, K_3_RhCl_6_, Na_2_PdCl_4_, Na_2_IrCl_6_·6H_2_O, Na_2_WO_4_·2H_2_O, K_2_RuCl_5_ 5H_2_O, and Na_2_MoO_4_·2H_2_O, various SACs (Ir, Ru, Pt, W, Mo, Rh, Au, and Pd) can be obtained using a similar approach. The PXRD results showed that the diffraction peaks of various eZIF‐M match well with ZIF‐8 and no diffraction of crystalline metal Pt was observed (Figure S11, Supporting Information). Besides, TEM, HAADF‐STEM, and corresponding EDS element mapping indicated that the target metal elements were uniformly distributed in each sample without obvious metal particle agglomeration (**Figure** **2**; Figure S12, Supporting Information). Uniformly dispersed atomic nature was also demonstrated by AC HAADF‐STEM (Figure 2). Metal single atoms were confirmed by isolated bright spots marked by white circles. In addition, the XPS results indicated that the metals in eZIF‐M carry a positive charge with an oxidation state (Figure S13, Supporting Information). To determine the amount of guest metal in ZIF‐8, inductively coupled plasma optical emission spectrometry (ICP‐OES) was performed and the results are shown in Table S2 (Supporting Information). Clearly, this one‐step dynamic electrosynthesis strategy extends a new pathway for the preparation of ZIF‐8‐supported single‐atom catalysts, providing a powerful platform for efficient catalytic reactions.^[^ ^45^, ^46^, ^47^ ^]^

### 2.2 Pyrolysis of eZIF‐Pt

**Figure 3:**
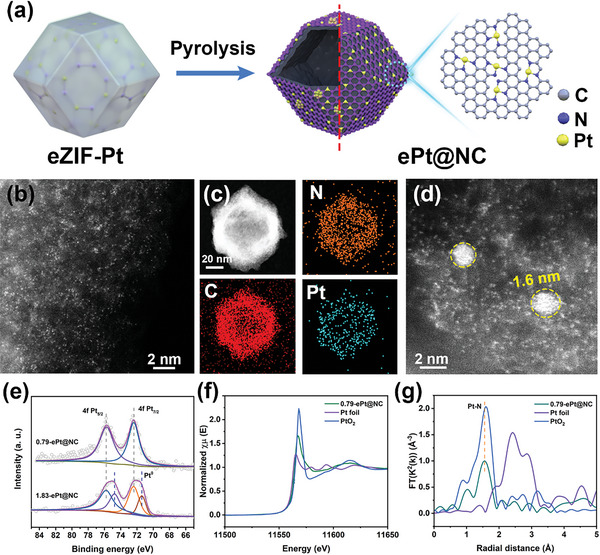
Preparation and characterizations of x‐ePt@NC. a) The schematic diagram of the synthesis procedure of x‐ePt@NC. b) AC HAADF‐STEM images, c) HAADF‐STEM and corresponding EDS element mapping images of the 0.79‐ePt@NC. d) AC HAADF‐STEM images of 1.83‐ePt@NC. e) High‐resolution Pt 4f XPS spectra of 0.79‐ePt@NC and 1.83‐ePt@NC. f) Normalized XANES spectra and g) k^2^‐weighted FT spectra from EXAFS at the Pt L_3_‐edge of 0.79‐ePt@NC, PtO_2_, and Pt foil.

To further explore the downstream application of the electrochemically synthesized catalysts and extend the scenario of the eZIF‐M, we pyrolyzed eZIF‐Pt to remove the Zn species and carbonize the organic ligands thereby fabricating nitrogen‐doped carbon‐supported Pt sites (x‐ePt@NC, x represents the weight percent by ICP‐OES) (**Figure** **3**a). During the pyrolysis process, the pre‐coordinated structure of Pt and the porous skeleton of ZIF‐8 effectively prevent the agglomeration of Pt atoms. As shown in Figure S14 (Supporting Information), the PXRD pattern of 0.79‐ePt@NC showed a broad C (002) peak, and no peak signal was observed for the crystalline metal Pt. Nitrogen‐doped carbon (NC) was further confirmed by Raman spectroscopy (Figure S15, Supporting Information) and XPS indicated that the dominant nitrogen species on NC were pyrrolic and pyridinic nitrogen (Figure S16, Supporting Information). SEM and TEM were performed to study the morphology and phase composition of the catalysts after pyrolysis. As shown in Figures S17 and S18 (Supporting Information), the 0.79‐ePt@NC shrinks into a hollow structure and no Pt crystal particles were observed, which was consistent with the results observed by selected area electron diffraction (Figure S19, Supporting Information). N_2_ sorption isotherms of 0.79‐ePt@NC showed that hollow NC possesses a large specific surface area (1188.1 m^2^ g^−1^) with abundant micropores, which facilitates the transport of active substances and charges during the catalytic process (Figure S20, Supporting Information). Additionally, Pt single atoms (bright spots) on 0.79‐ePt@NC can be clearly observed by AC HAADF‐STEM (Figure 3b). The HAADF‐STEM and corresponding EDX elemental mapping results also confirm that Pt, N, and C were uniformly distributed on 0.79‐ePt@NC (Figure 3c; Figure S21, Supporting Information). However, as the Pt loading amount increases (1.83‐ePt@NC), some of the isolated Pt atoms were induced to form nanoclusters ≈2.0 nm (Figure 3d; Figure S22, Supporting Information). The high‐resolution XPS spectra of 0.79‐ePt@NC showed two peaks centered at 72.38 and 75.78 eV attributed to Pt 4f_7/2_ and Pt 4f_5/2_ respectively, indicating the Pt species was close to +2 valence (Figure 3e). In contrast, 1.83‐ePt@NC showed subpeaks of Pt^0^ associated with nanoclusters at 71.4 and 74.7 eV, which was consistent with the results observed in AC HAADF‐STEM.

The detailed information on the electronic state and chemical environment of Pt single atoms were further investigated by XAS. As shown by normalized XANES in Figure 3f, the white line peak intensity of 0.79‐ePt@NC was between those of Pt foil and PtO_2_, suggesting that Pt was positively charged.^[^ ^42^ ^]^ Precisely, the average oxidation state of Pt single atoms was calculated as +2.4 based on the E_0_ value, which was consistent with the XPS result (Figure S23, Supporting Information).^[^ ^48^ ^]^ The coordination configuration of Pt single atoms was further revealed by EXAFS of Pt L_3_‐edge. As shown in Figure 3g, a major peak of ≈1.56 Å was assigned to the Pt─N bond in 0.79‐ePt@NC, and no characteristic peak for the Pt─Pt bond was observed. The EXAFS WT analysis with high resolution in both R‐ and *k*‐spaces further indicated an intensity maximum at ≈5.4 Å^−1^ attributed to the Pt─N bond (Figure S24, Supporting Information). Furthermore, the coordination configuration was investigated by quantitative least‐squares EXAFS curve‐fitting analysis (Figures S25 and S26, Supporting Information). The fitting results in R and *k*‐space showed that the coordination number of the Pt−N in the first coordination shell layer for Pt single atoms was ≈3.0 (Table S3, Supporting Information).

### 2.3 Electrocatalytic HER Performance of ePt@NC

**Figure 4:**
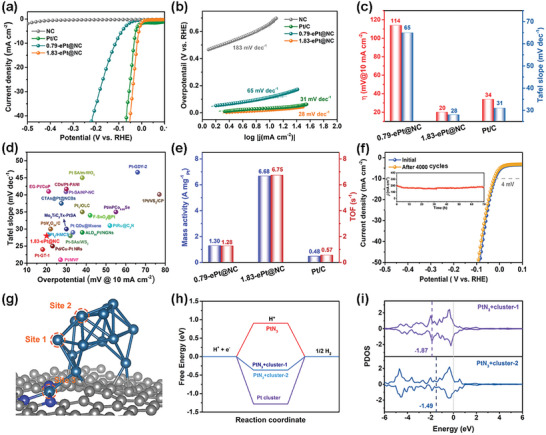
Electrocatalytic HER performance and DFT calculations. a) LSV curves of the 0.79‐ePt@NC, 1.83‐Pt@NC, commercial Pt/C, and NC. b) The corresponding Tafel slope originated from LSV curves. c) Comparison of overpotentials at 10 mA cm^−2^ and Tafel slopes. d) Comparison of the HER activity for 1.83‐Pt@NC with reported catalysts, originating from Table S4 (Supporting Information). e) Mass activity and TOF values at an overpotential of 100 mV. f) Stability test of 1.83‐Pt@NC through cyclic potential scanning (inset: chronoamperometry method). g) Atomic configuration of Pt cluster/PtN_3_ (The dashed circles represent different H adsorption sites). h) The ∆*G*_H*_ of H adsorption on different sites in (g). i) The PDOS plots of Pt cluster/PtN_3_.

To evaluate the electrocatalytic HER activity of x‐ePt@NC, the obtained catalyst was examined in an acidic electrolyte (0.5 m H_2_SO_4_) using a standard three‐electrode system. The commercial Pt/C (20 wt.%) was also tested under the same conditions for comparison. As seen from the linear sweep voltammetry (LSV) curve in **Figure** **4**a, the 1.83‐ePt@NC exhibited a very small overpotential of ≈20 mV at a current density of 10 mA cm^−2^, which is superior to that of commercial Pt/C (34 mV) (Figure 4c). In contrast, 0.79‐ePt@NC requires a much higher overpotential of 114 mV to reach 10 mA cm^−2^. From the Tafel slope, the 1.83‐ePt@NC catalyst was as low as 28 mV dec^−1^, comparable to commercial Pt/C (31 mV dec^−1^), indicating a Volmer–Tafel mechanism and that the Tafel step was the rate‐limiting step (Figure 4b,c).^[^ ^49^ ^]^ The outstanding HER performance of 1.83‐ePt@NC was superior to most of the recently reported Pt‐based catalysts in acidic media (Figure 4d; Table S4, Supporting Information). In comparison, 0.79‐ePt@NC has a much higher Tafel slope of 65 mV dec^−1^, indicating slower reaction kinetics, which was further confirmed by the electrochemical impedance spectra. As shown in the Nyquist plot (Figure S27, Supporting Information), 1.83‐Pt@NC has a smaller arc radius in the high‐frequency region compared to 0.79‐Pt@NC, indicating a lower charge transfer resistance. Obviously, the low charge transfer resistance of 1.83‐Pt@NC was attributed to the formation of active Pt cluster sites and possible single‐atoms and cluster synergies, which will be further discussed in the calculation section.

To further characterize the activity of the prepared catalysts, the mass activities of Pt in commercial Pt/C, 0.79‐ePt@NC, and 1.83‐ePt@NC catalysts were compared by normalizing the LSV curves. As shown in Figure 4e and Figure S28 (Supporting Information), the 1.83‐ePt@NC exhibited absolute dominance at different potentials. The mass activity of 1.83‐ePt@NC was as high as 6.68 A mg^−1^_Pt_ at −0.1 V, which was 5.14 and 13.92 times greater than that of 0.79‐ePt@NC and commercial Pt/C, respectively. Moreover, the turnover frequency (TOF) of catalysts was calculated to further evaluate their intrinsic activities. The results indicated that 1.83‐ePt@NC showed higher TOF compared to 0.79‐ePt@NC and commercial Pt/C with the applied potentials (Figure 4e; Figure S29, Supporting Information). In particular, the TOF values at −0.1 V for 1.83‐ePt@NC (6.75 s^−1^) were 5.27 and 11.84 times higher than those for the 0.79‐Pt@NC and commercial Pt/C catalysts (according to the CO stripping experiment, Figure S30, Supporting Information), respectively.

In addition to catalytic activity, stability was also a crucial property in practical applications. The stability of the 1.83‐ePt@NC catalyst was estimated by accelerated durability tests and potential‐constant electrolysis. As shown in Figure 4f, the polarization curve exhibited only a marginal negative shift after 4000 cycles. In addition, the current density of 1.83‐ePt@NC did not decay obviously for 70 h at high current density (≈200 mA cm^−2^)(inset of Figure 4f). As a compare comparison, the current density of 0.79‐Pt@NC showed a slight decay during constant‐potential electrolysis (Figure S31, Supporting Information). The XPS, TEM, and HAADF‐STEM of catalysts after the stability test indicated that the catalyst structure changed negligibly and that no large nanoparticles formed (Figures S32–S34, Supporting Information). Overall, the excellent electrical/intrinsic activity and long‐term durability endow 1.83‐ePt@NC as a potential HER electrocatalyst for practical applications.

### 2.4 Density Functional Theory Calculations

To gain a deeper insight into the origins of distinguished HER activity for Pt cluster/single atoms, theoretical studies were performed based on density functional theory (DFT) calculations. According to EXAFS and HAADF‐STEM, the Pt cluster was constructed on the PtN_3_‐graphene surface aiming to simulate the atomic structure of 1.83‐ePt@NC (Figure 4g). The active site of 1.83‐ePt@NC was explored first, while the pure Pt cluster and PtN_3_ were further investigated for comparison. For Pt cluster/PtN_3_ (Figure 4g; Figure S35, Supporting Information), there were three possible sites for H adsorption, i.e., the PtN_3_ site (site 3), and two possible Pt atoms of Pt cluster (sites 1 and 2). However, after structure relaxation, there was no stable adsorption of H on site 3. Therefore, the active sites for HER of Pt cluster/PtN_3_ were mainly on the Pt cluster. The Gibbs free energy changes on different sites, including pure PtN_3_ and Pt cluster, were calculated. It was evident from Figure 4h that the HER activity would be improved on Pt cluster/PtN_3_, i.e., Δ*G*_H_ = 0.36 and 0.35 eV for 1 and 2 sites, respectively, compared with pure PtN_3_ (0.90 eV) and Pt cluster (−1.28 eV)^[^ ^50^ ^]^ To further explore the physical mechanism of the improved catalytic property, the electronic structures of active Pt atom was estimated (Figure 4i; Figure S36, Supporting Information). The calculated projected density of states (PDOS) indicated that the active Pt d‐band center of Pt cluster/PtN_3_ was optimized to a suitable site (−1.87 eV for site 1, −1.49 eV for site 2), compared with pure PtN_3_ (−2.28 eV) and Pt cluster (−1.28 eV). Therefore, the H adsorption became stronger (weaker) on the Pt cluster/PtN_3_ than the pure PtN_3_ (Pt cluster), suggesting that PtN_3_ could effectively regulate the electronic structures of the Pt cluster and improve the HER activities. On the other hand, for the differential charge (Figure S37, Supporting Information), the negligible transferred charge (0.07 e) was observed, indicating that the introduced PtN_3_ did not highly change the electron number of Pt clusters, which was also favorable for the HER. Hence, the synergy between Pt clusters and single atoms in 1.83‐ePt@NC was a major reason for the significant enhancement of HER activity.

## 3 Conclusion

In summary, the electrochemical synthetic strategy described here was a straightforward and promising avenue to manufacture SACs. Such a universal synthesis method only required the replacement of different metal precursors to prepare various SACs. Moreover, the as‐derived Pt single atoms/cluster catalysts generated a superior synergistic effect, endowing excellent electrocatalytic HER activity and stability. It should also be noted that as economical, sustainable, and facile routes, electrochemical synthesis ensured the promising potential for scalable preparation. Hence, this work not only enriches the single‐atom synthesis methodology but also extends the implications of electrochemical synthesis, opening up new avenues for the design of advanced electro‐synthesized catalysts.

## Conflict of Interest

The authors declare no conflict of interest.

## Supporting information

Supporting Information

## Data Availability

The data that support the findings of this study are available in the supplementary material of this article.
