# Supplementary material for: A Universal Electrochemical Synthetic Strategy for the Direct Assembly of Single‐Atom Catalysts
Source: Adv Sci (Weinh). 2023 Oct 12;10(34):2304656. doi: 10.1002/advs.202304656 (PMC10700173; doi:10.1002/advs.202304656)
Supplement: Supplementary file 1 — Supporting Information [file ADVS-10-2304656-s001.pdf]

ADVANCED SCIENCE
Open Access

Supporting Information

for *Adv. Sci.*, DOI 10.1002/advs.202304656

A Universal Electrochemical Synthetic Strategy for the Direct Assembly of Single-Atom Catalysts

Ya-Kun Lv, Kun Wang, Wen-Yan Sun, Peng Peng and Shuang-Quan Zang**

Supporting Information

A universal electrochemical synthetic strategy for the direct assembly of single-atom catalysts

Ya-Kun Lv, Kun Wang, Wen-Yan Sun, Peng Peng, Shuang-Quan Zang**

Experimental Section

Chemicals. All chemicals and reagents used in this research, including 2-methylimidazole($C_4H_6N_2$), potassium chloride (KCl), methanol, potassium hexachlororhodate (K_2PtCl_6), gold (III) chloride hydrate ($HAuCl_4·xH_2O$), Tripotassium hexachlororhodium (III) (K_3RhCl_6), sodium tetrachloropalladate (II) (Na_2PdCl_4), sodium hexachloroiridate (IV) hexahydrate ($Na_2IrCl_6·6H_2O$), sodium tungstate dehydrate ($Na_2WO_4·2H_2O$), potassium pentachlororuthenate (III) hydrate ($K_2RuCl_5·xH_2O$), and sodium molybdate dehydrate ($Na_2MoO_4·2H_2O$) were purchased commercially and used without further purification. The hydrophilic carbon cloth was treated with a mixture of 10% (*v/v*) nitric acid and 10% (*v/v*) sulfuric acid and then rinsed with deionized water three times before use. High-purity zinc foil (Zn≧99.99%) was sonicated sequentially in acetone and ethanol before use and then washed with deionized water. All chemicals used in the experiment are analytical-grade reagents.

Electrochemical synthesis of ZIF-8-supported single atoms (eZIF-M). eZIF-M (M = Pt, Pd, Ir, Ru, Rh, Au, Mo, W) was synthesized by a cyclic voltammetry method. The synthesis was conducted in a single cell using a two-electrode system. A 2×2.5 cm^{-2} Zn sheet was used as the working electrode and a 2×2.5 cm^{-2} carbon cloth was used as the auxiliary electrode. The solution of methanol/H_2O (15:17 *v/v*, 32 mL) contains 0.26 mM metal precursors, 42 mM KCl, and 460 mM 2-Methylimidazole, and the resulting clear solution was used as electrolyte. K_2PtCl_6, $HAuCl_4·xH_2O$, K_3RhCl_6, Na_2PdCl_4, $Na_2IrCl_6·6H_2O$, $Na_2WO_4·2H_2O$, K_2RuCl_5 xH_2O, and $Na_2MoO_4·2H_2O$ were used as the metal precursors for Pt, Au, Rh, Pd, Ir, W, Ru, and Mo, respectively. The electrochemical synthesis was carried out by 200 cycles of cyclic voltammetry with a sweeping rate of 100 mV s^{-1}. The potential windows were -3.2-3.6 V for Pt, Au and Rh, -2.0-3.2 V for Pd and Ir, -2.6-3.2 V for Ru and W, as well as -3.8-3.6 V for Mo, respectively. After the reactions, the eZIF-M was collected by centrifugation, washed three times with water and methanol, and dried at 60 °C. Pure ZIF-8 (eZIF-8) was synthesized in the same way as described above (the potential window is -3.2-3.6 V), except that no metal precursor was added.

Synthesis of x-ePt@NC. According to the above method, eZIF-Pt with different Pt loads was first obtained by adding K_2PtCl_6 with 0.26 and 0.1 mM, respectively. The powder of eZIF-Pt was placed in a tube furnace and heated to 950 °C for 3 h at a heating rate of 7 °C min^{-1} under Ar gas. The prepared catalyst was named x-ePt@NC, with x representing the Pt loading.

Material characterization. Powder X-ray diffraction patterns (PXRD) were collected on a Rigaku MiniFlex 600 diffractometer with CuKa radiation (λ = 1.5418 Å) at 30 kV. The

compositions and morphologies of the catalysts were observed using scanning electron microscopy (SEM, Zeiss Sigma 500) and transmission electron microscopy (TEM, FEI TalosF200S). The high-angle annular dark-field scanning transmission electron microscopy (HAADF-STEM) and Energy-dispersive X-ray Spectra (EDS) element mapping were acquired using an aberration-corrected JEOLJEMARM300F TEM/STEM and transmission electron microscopy (TEM, FEI TalosF200S), respectively. The metal contents were determined by SHIMADZU ICPE-9820 inductively coupled plasma optical emission spectrometry (ICP-OES). X-ray photoelectron spectroscopy (XPS) was measured on a VG Scientific ESCALAB 250 photoelectron spectrometer equipped with a 300 W aluminum target radiation source (Al Kα). The Raman spectra were tested using a LabRam HR confocal Raman spectrometer with a laser excitation wavelength of 532 nm. Dynamic light scattering (DLS) measurements were performed on a Horiba SZ-100 Nanoparticle Size Analyzer. The nitrogen sorption isotherms were measured using the Belsorp Max automatic volumetric adsorption system. The specific surface areas and pore size distribution were calculated by using the Brunauer–Emmett–Teller (BET) equation and the non-linear density functional theory (NL-DFT) model. Fourier transform infrared (FT-IR) spectra were performed on a Bruker ALPHAⅡFT-IR spectrometer.

XAS measurements. Pt L_3-edge analysis was performed with Si(311) crystal monochromators at the BL14W1 beamlines at the Shanghai Synchrotron Radiation Facility (SSRF) (Shanghai, China). Before the analysis at the beamline, samples were pressed into thin sheets with 1 cm in diameter and sealed using Kapton tape film. The XAFS spectra were recorded at room temperature using a 4-channel Silicon Drift Detector (SDD) Bruker 5040. Pt L_3-edge extended X-ray absorption fine structure (EXAFS) spectra were recorded in fluorescence mode. Negligible changes in the line-shape and peak position of Pt L_3-edge XANES spectra were observed between two scans taken for a specific sample. The XAFS spectra of these standard samples (Pt foil and PtO_2) were recorded in transmission mode.

Data reduction, data analysis, and EXAFS fitting were applied through Athena and Artemis software.[1] The energy calibration of the sample was conducted through a standard Pt foil, which as a reference was simultaneously measured. For EXAFS modeling, the global amplitude EXAFS (CN, R, σ^2 and ΔE_0) were obtained by nonlinear fitting, with least-squares refinement, of the EXAFS equation to the Fourier-transformed data in R-space, using Artemis software, EXAFS of the Pt foil was fitted and the obtained amplitude reduction factor S_0^2 value was set in the EXAFS analysis to determine the coordination numbers (CNs) in the scattering path in samples. The Debye-Waller factors and delta Rs were obtained based on the *guessing* parameters and constrained for Pt-N. Wavelet transformation (WT) was also employed using

the software package developed by Funke and Chukalina using Morlet wavelet with $\kappa = 10$, $\sigma = 1$.[2, 3]

Electrochemical measurements. The electrochemical tests in this paper are all completed using Shanghai Chenhua CHI 660E electrochemical workstation and standard three-electrode system. The Ag/AgCl electrode (built-in saturated potassium chloride electrolyte) was used as the reference electrode, and the graphite rod was used as the counter electrode. All measured electrode potentials were obtained by the formula E (RHE) = E (Ag/AgCl) + 0.199 + 0.059pH conversion. The working electrode adopts a glassy carbon electrode (GCE) whose surface was modified by catalysts, and its preparation method was as follows: the well-dispersed catalyst ink was prepared by ultrasonication with 5.0 mg catalyst, 490 μL C_2H_5OH and 10 μL Nafion solution (5 wt%) for 30 min. Then, 5 μL of the catalyst ink was pipetted onto the GCE surface (3 mm in diameter, S = 0.0706 cm^2). The loading amount of catalysts was 0.71 mg cm^{-2} on the GCE. A certain amount of prepared catalyst slurry was applied to the surface of the GCE and the catalyst-modified working electrode was obtained after drying at room temperature. The long-term stability of 1.83-ePt@NC and 0.79-ePt@NC catalysts was tested using the chronoamperometric method. In this procedure, catalysts (0.71 mg cm^{-2}) were pipetted onto the GCE (L-shaped, S = 0.0706 cm^2) surfaces and the applied potential was 0.3 V (vs. RHE).

Computational Details. The calculations were carried out using density functional theory with the PBEform of generalized gradient approximation functional (GGA).[4] The Vienna ab-initio simulation package (VASP)[5-8] was employed. The plane wave energy cutoff was set as 400 eV. The Fermi scheme was employed for electron occupancy with an energy smearing of 0.1 eV. The first Brillouin zone was sampled in the Monkhorst−Pack grid.[9] The 3×3×1 k-point mesh for the surface calculation. The energy (converged to 1.0 $\times 10^{-6}$ eV/atom) and force (converged to 0.01eV/Å) were set as the convergence criterion for geometry optimization. The spin polarization was considered in all calculations.

Model. The graphene (001) surface was employed as the support for the PtN_3 and Pt clusters. For Pt clusters, the Pt_{13} will be used, while the defect site near the PtN_3 will be selected as the adsorption site for Pt_{13}.In structural optimization calculations, all atoms were allowed to relax. A vacuum layer as large as 15 Å was used along the c direction normal to the surface to avoid periodic interactions.[10, 11]

For HER, The Gibbs free-energy change (ΔG_{ads}) of H on the catalyst is defined as follows:

$$\Delta G_{ads} = \Delta E_{ads} + \Delta E_{ZPE} - T\Delta S$$

where ΔE_{ads} is the adsorption energy of the atomic H on the catalyst, and ΔE_{ZPE} is the difference in zero-point energy between the adsorbed hydrogen and hydrogen in the gas phase. ΔS is the entropy change of one H atom from the absorbed state to the gas phase. Since the H atom is binding on the surface, the entropy of the adsorbed hydrogen can be negligible. Therefore, the ΔS can be estimated by -1/2×S_0, in which S_0 is the standard entropy of H_2 with gas phase at a pressure of 1 bar and pH = 0 at 300 K

The ΔE_{ads} is defined as follows:

$$\Delta E_{ads} = E_{H/slab} - (E_{slab} + \frac{1}{2}E_{H_2})$$

where the $E_{H/slab}$ is the total energy of the H atom on catalysts, E_{slab} is the total energy of the catalyst, and E_H is the energy of the H atom reference to the gas H_2. The first two terms are calculated with the same parameters. The third term is calculated by setting the isolated H_2 in a box of 12 Å×12 Å×12 Å.

Calculation of turnover frequency (TOF) and mass activity. The TOF value was calculated according to the previous report, and the detail was described below: [12]

$$\mathrm{TOF} = \frac{\#\ total\ hydrogen\ turnovers\ per\ geometric\ area}{\#\ Active\ sites\ per\ geometric\ area}$$

The number of total hydrogen turnovers was calculated from the current density extracted from the LSV polarization curve according to the following equation:

$$\begin{aligned}&\text{Total hydrogen turnovers}\\ &= \left(|j|\frac{\mathrm{mA}}{\mathrm{cm^{-2}}}\right)\left(\frac{1\ \mathrm{C/s}}{1000\ \mathrm{mA}}\right)\left(\frac{1\ \mathrm{mol/e}}{96485\ \mathrm{C}}\right)\left(\frac{1\ \mathrm{mol}}{2\ \mathrm{mol/e}}\right)\left(\frac{6.002\times10^{23}\ \text{molecular}\ \mathrm{H_2}}{1\ \mathrm{mol\ H_2}}\right)\\ &= 3.12\times10^{15}\,\frac{\mathrm{H_2/s}}{\mathrm{cm^2}}\ \text{per}\ \frac{\mathrm{mA}}{\mathrm{cm^2}}\end{aligned}$$

The number of active sites in x-ePt@NC was calculated from the mass loading on the GCE, assuming each Pt center accounts for one active site:

$$\begin{aligned}&\text{Active sites}\\ &= \left(\frac{\text{catalyst loading per geometric area}\ \left(\mathrm{x}\frac{\mathrm{g}}{\mathrm{cm^2}}\times \mathrm{Pt\ wt\%}\right)}{\mathrm{Pt\ M_w}\ \left(\frac{\mathrm{g}}{\mathrm{mol}}\right)}\right)\left(\frac{6.022\times10^{23}\ \text{Pt atoms}}{1\ \text{mol Pt}}\right)\end{aligned}$$

For commercial Pt/C, the active sites were calculated according to the following equation:

$$\text{Active sites} = \frac{Q_s/A_{disk}}{\mathrm{F}}$$

where A_{disk} was the area of GCE, F was the Faraday constant, and Q_s was the amount of transferred charge calculated from the integral area of the CO striping region of catalysts in 0.5 M H_2SO_4. Before the test, the electrolyte was purged by high-purity N_2 gas for 20 min. Subsequently, high-purity CO was bubbled into the electrolyte for 20 min to achieve maximum coverage of CO on the metal surface. Finally, the dissolved CO in the electrolyte was purged out by bubbling high-purity N_2 gas for 15 min.

The mass activity is derived from the current density that was normalized by the mass loading, which was calculated by the below equation:[12]

$$\text{Mass activity} = \frac{j \times A_{disk}}{\text{The mass of Pt on the electrode}}$$

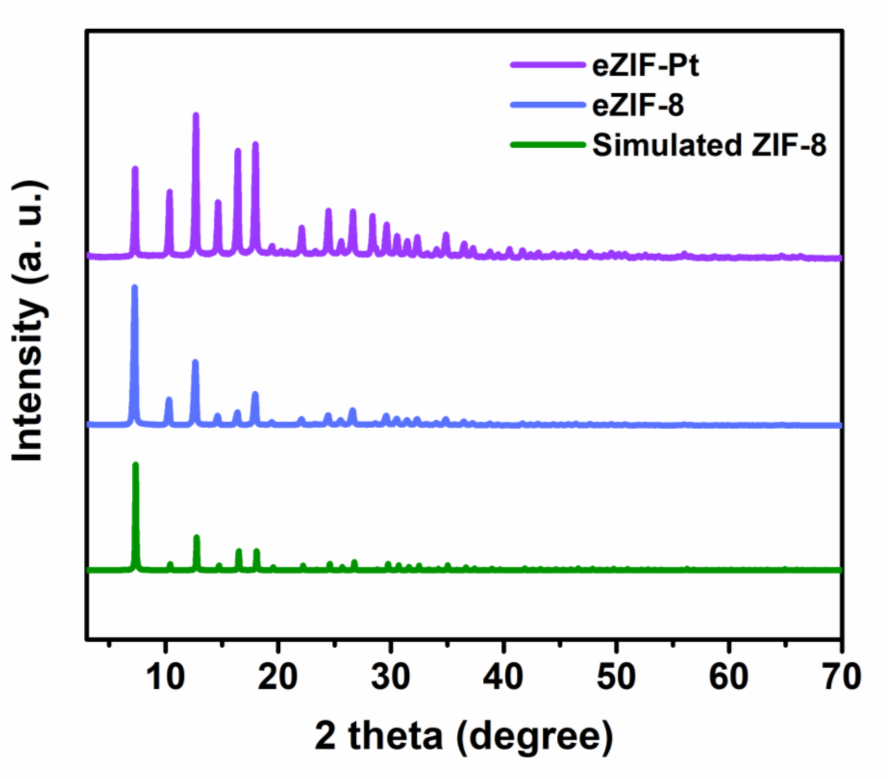

Figure S1. PXRD patterns of eZIF-Pt, eZIF-8 and simulated ZIF-8.

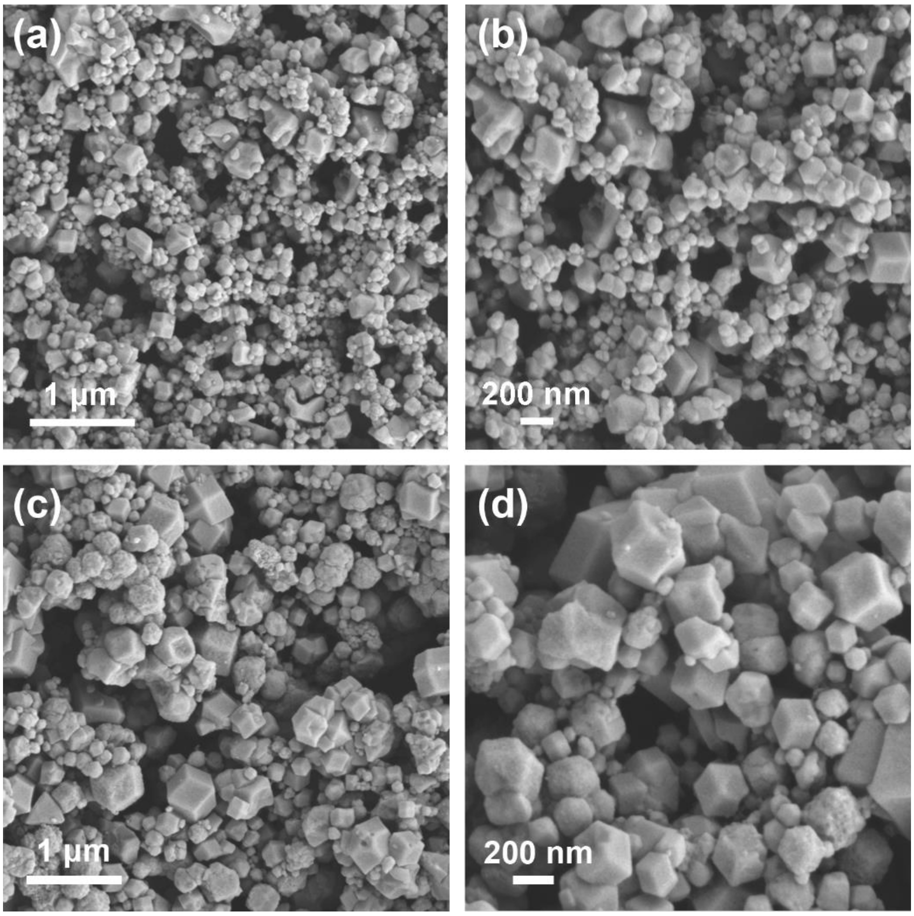

Figure S2. SEM images of (a, b) eZIF-8 and (c, d) eZIF-Pt.

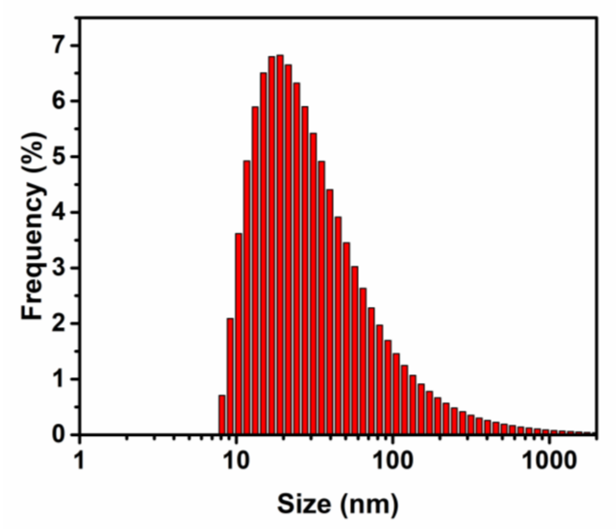

Figure S3. DLS measurement of eZIF-Pt.

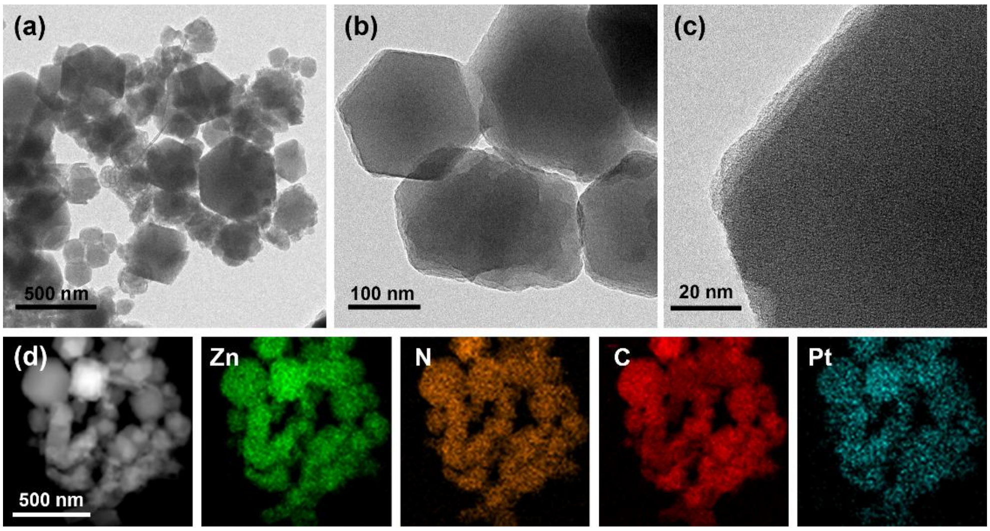

Figure S4. TEM images of different magnifications of (a-c) eZIF-Pt and (d) HAADF STEM and corresponding EDS mapping of low magnification.

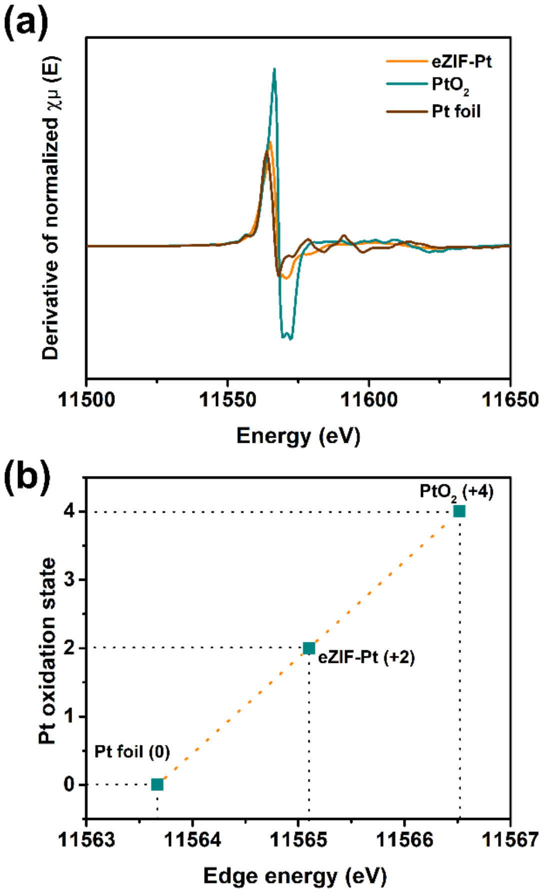

Figure S5. (a) First derivatives of Pt L-edge XANES regions of Pt foil, PtO_2, and eZIF-Pt. (b) The fitted average oxidation states of Pt from XANES spectra.

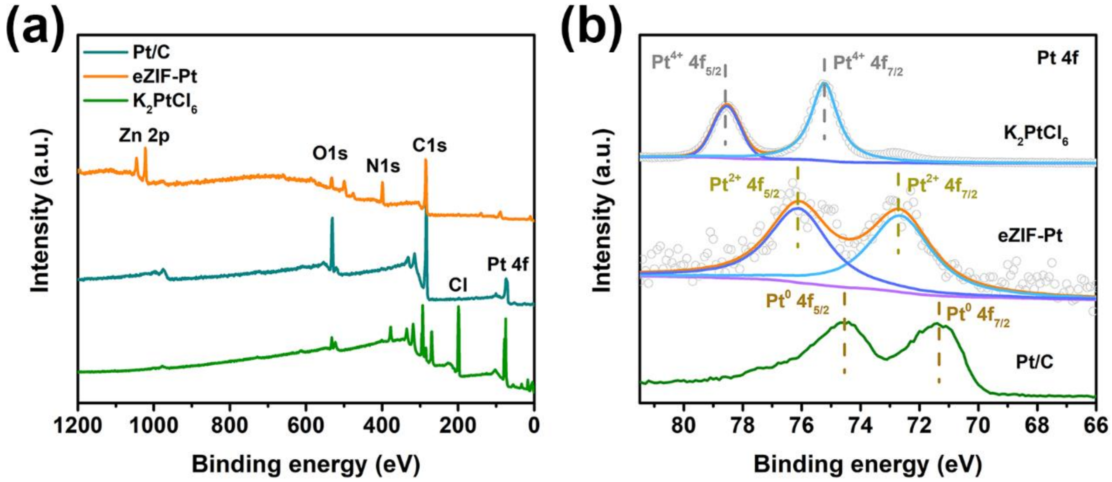

Figure S6. (a) XPS survey spectra and (b) high-resolution Pt 4f XPS spectra of eZIF-Pt, K_2PtCl_6, and commercial Pt/C.

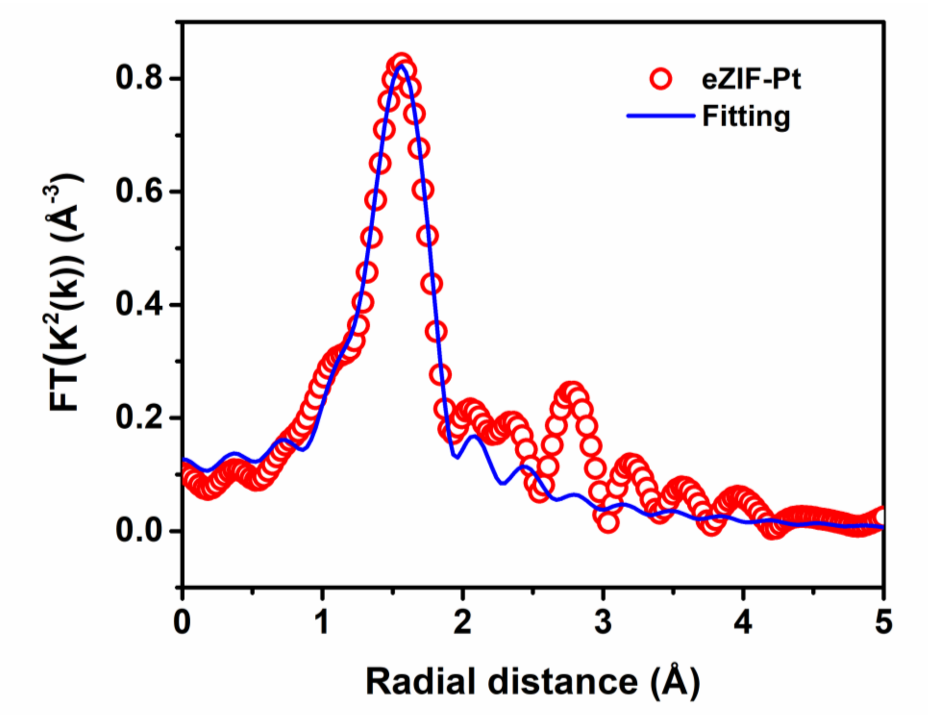

Figure S7. Experimental and fitting EXAFS curves for eZIF-Pt in R space.

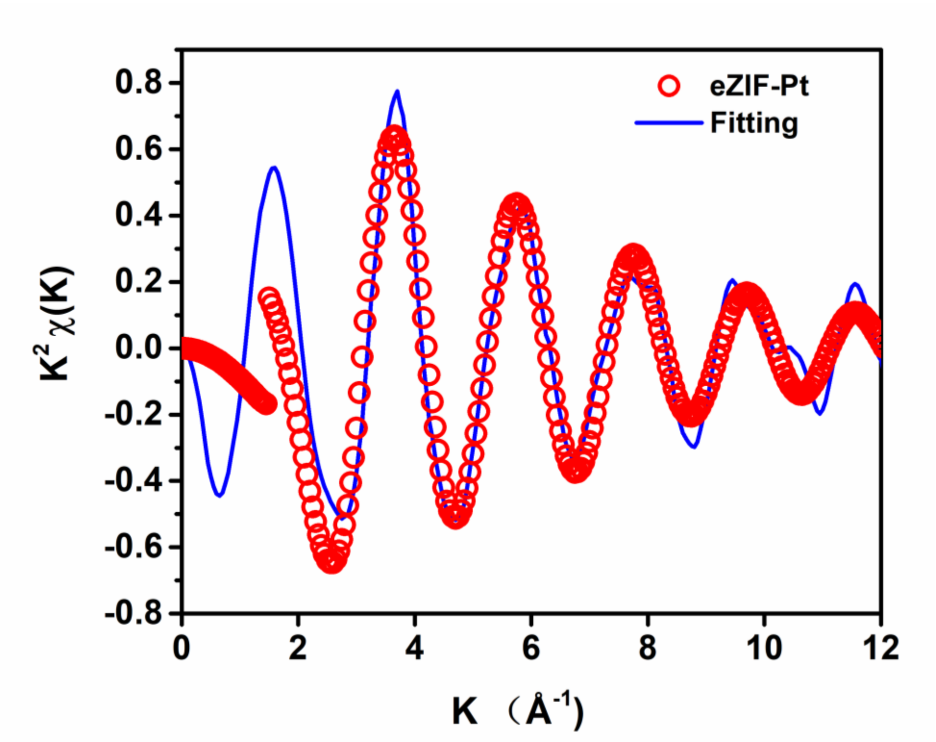

Figure S8. EXAFS fitting curves of eZIF-Pt at the K space.

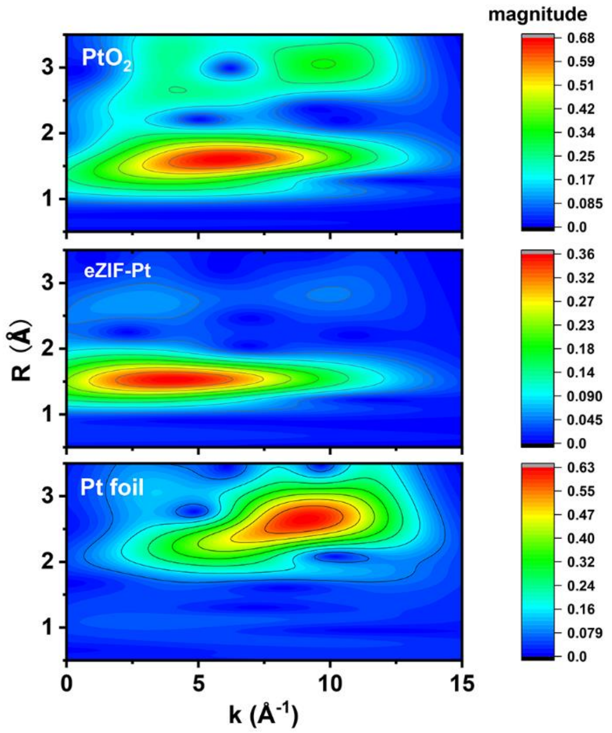

Figure S9. The wavelet transform of the eZIF-Pt, Pt foil and PtO_2.

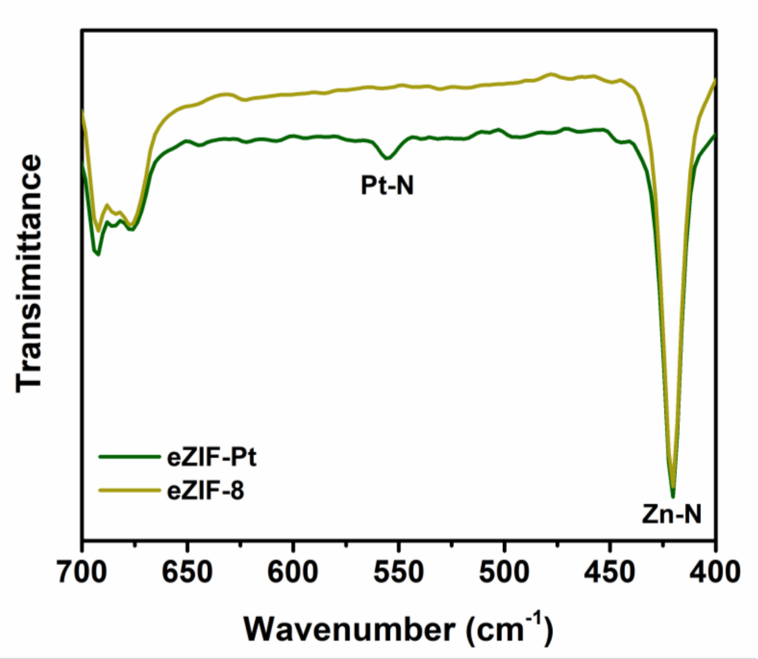

Figure S10. FT-IR spectra of eZIF-Pt.

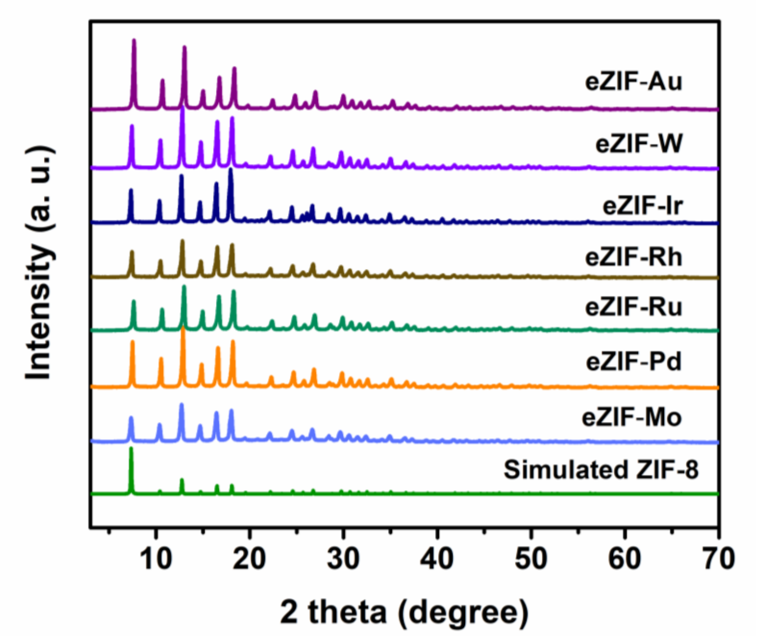

Figure S11. PXRD pattern of eZIF-M.

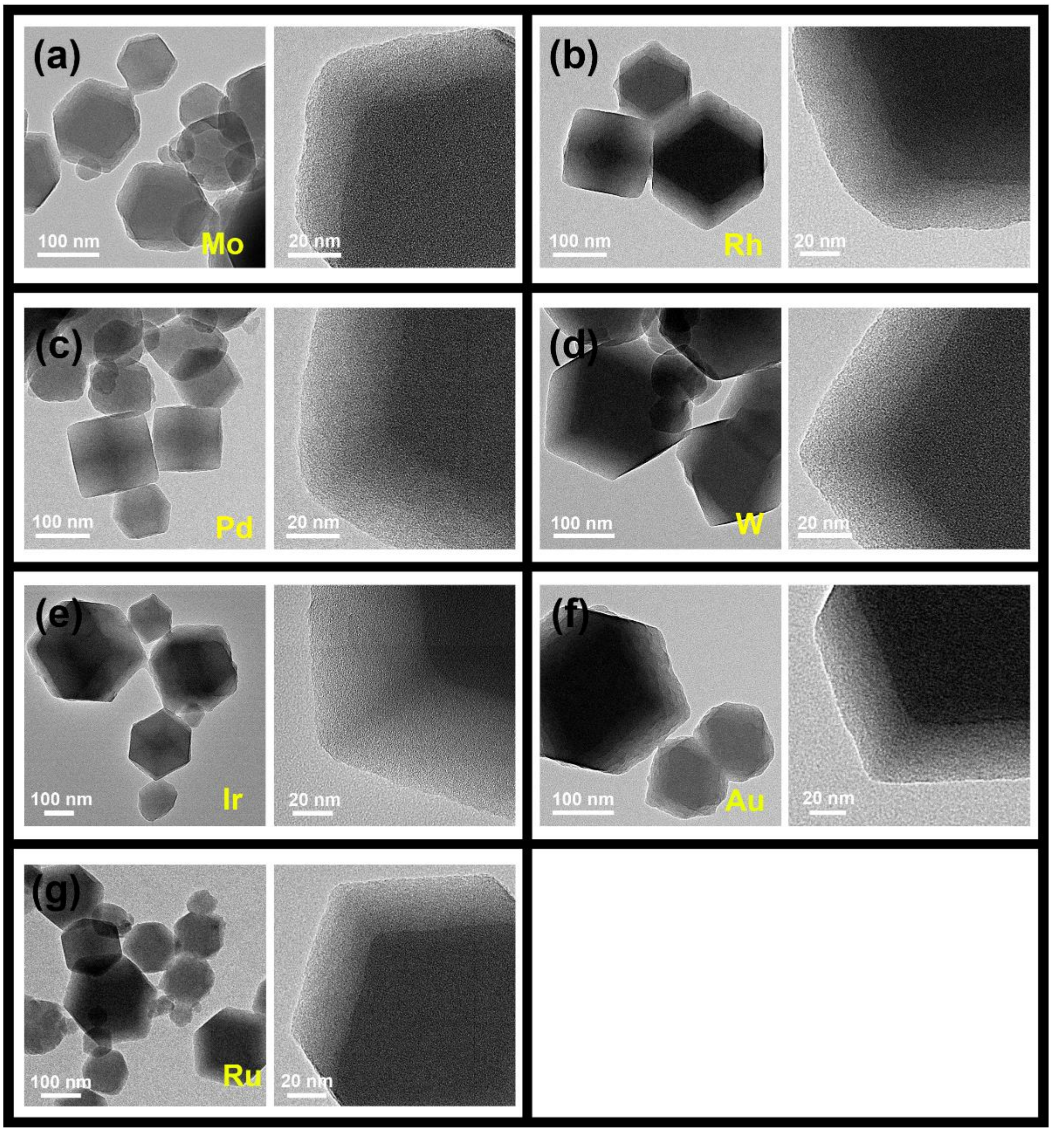

Figure S12. TEM images of eZIF-M.

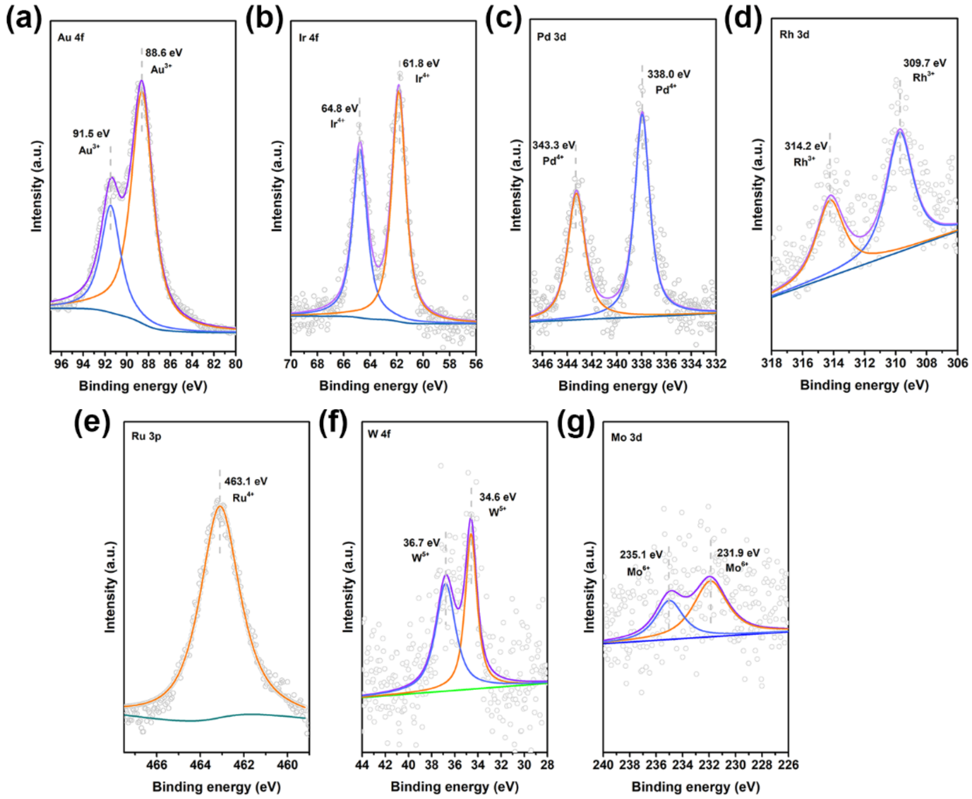

Figure S13. High-resolution XPS spectra of (a) Au 4f, (b) Ir 4f, (c) Pd 3d, (d) Rh 3d, (e) Ru 3p, (f) W 4f, and (g) Mo 3d.

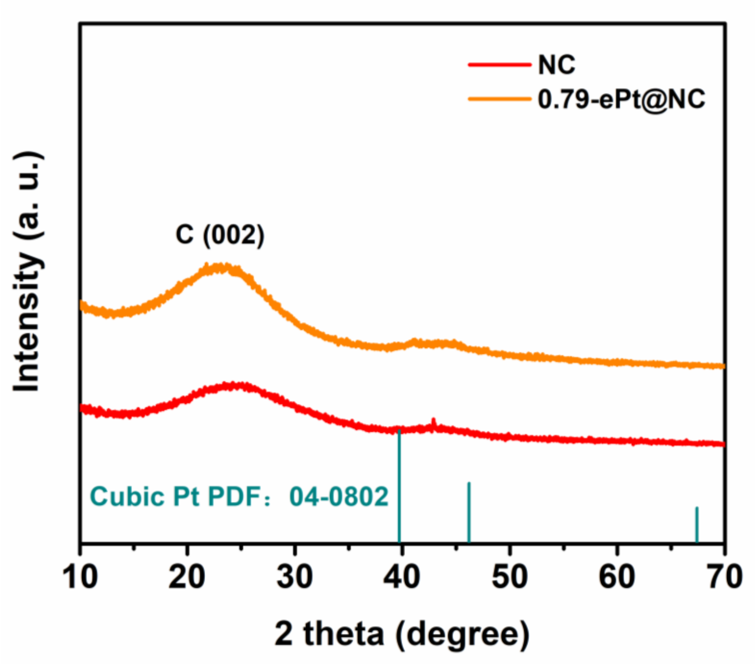

Figure S14. PXRD pattern of NC and 0.79-ePt@NC.

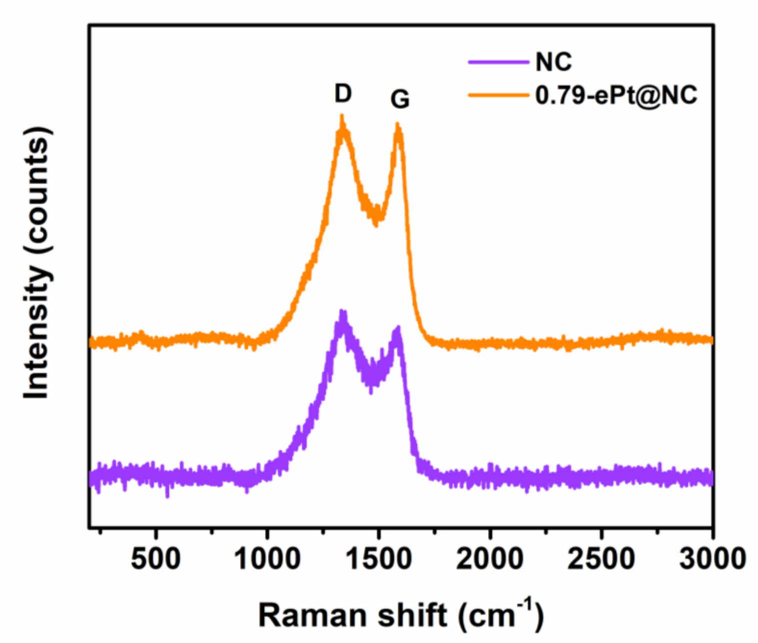

Figure S15. Raman spectra of NC and 0.79-ePt@NC.

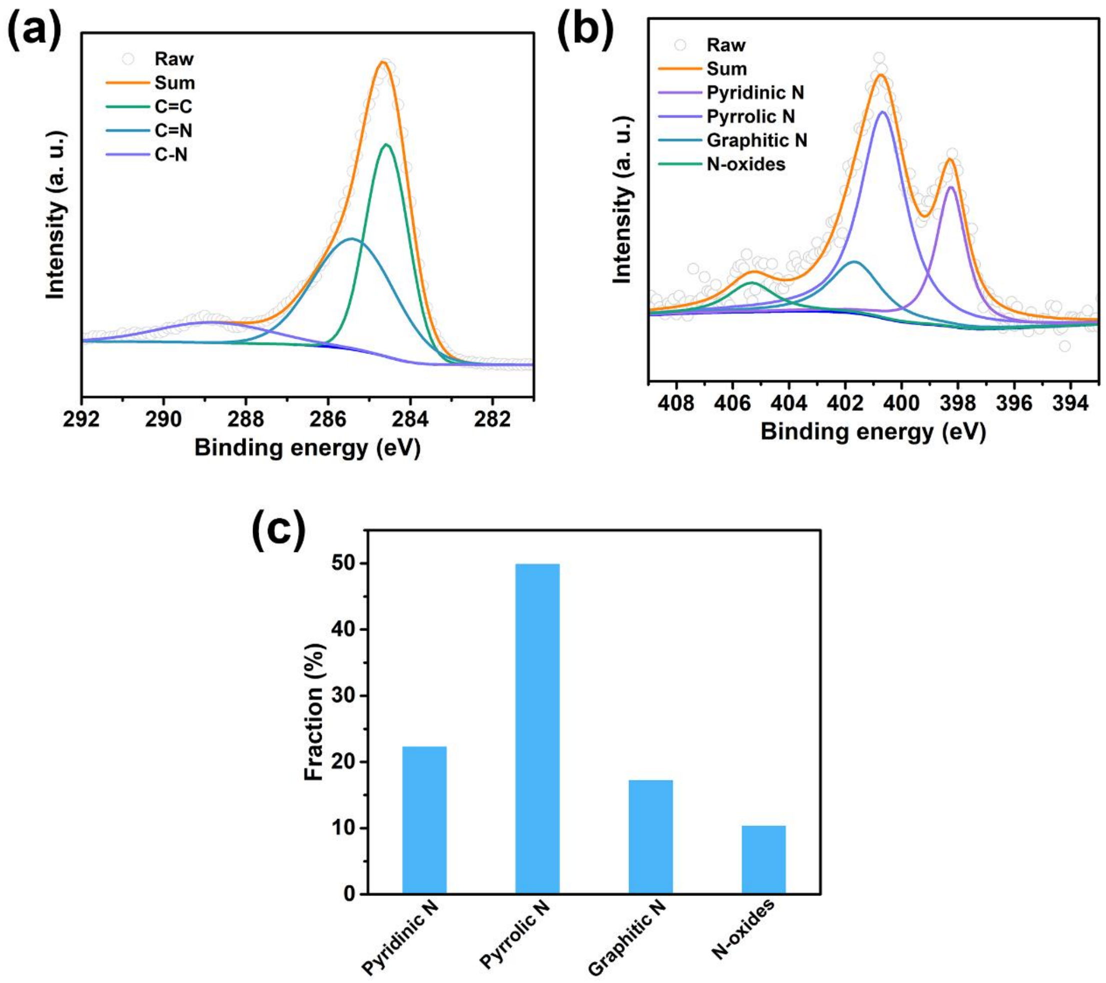

Figure S16. XPS spectra of 0.79-ePt@NC for (a) C 1s regions, (b) N 1s regions, and (c) the corresponding N content.

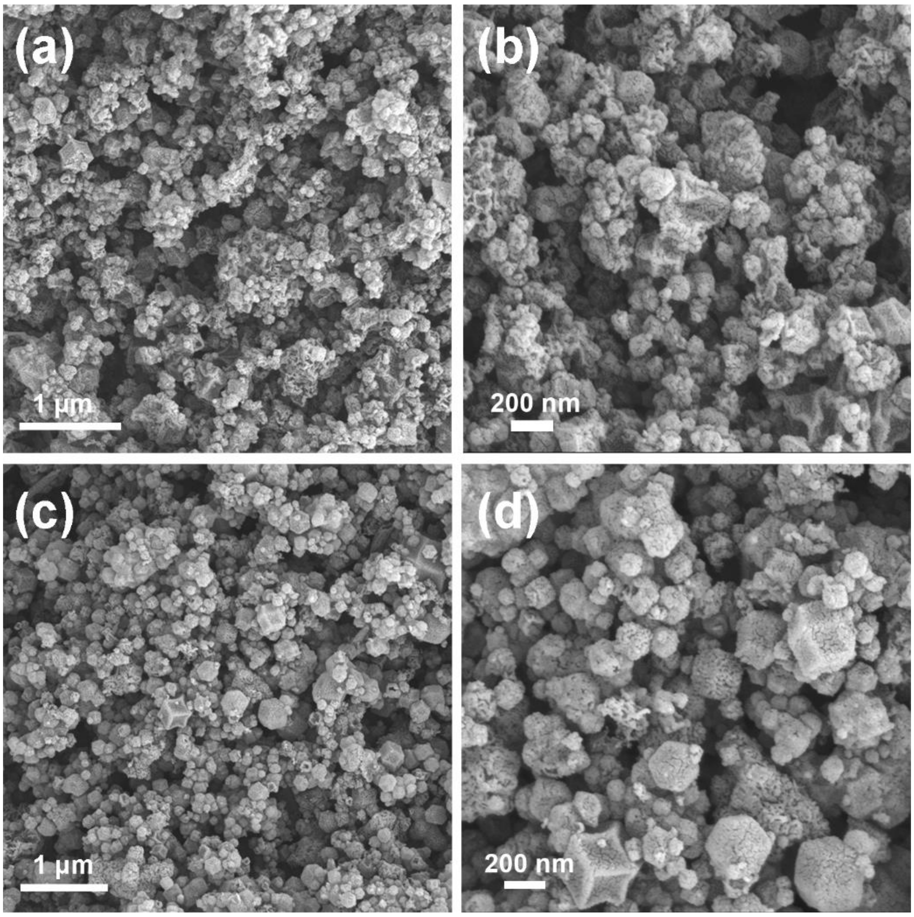

Figure S17. SEM images of (a, b) NC and (c, d) 0.79-ePt@NC.

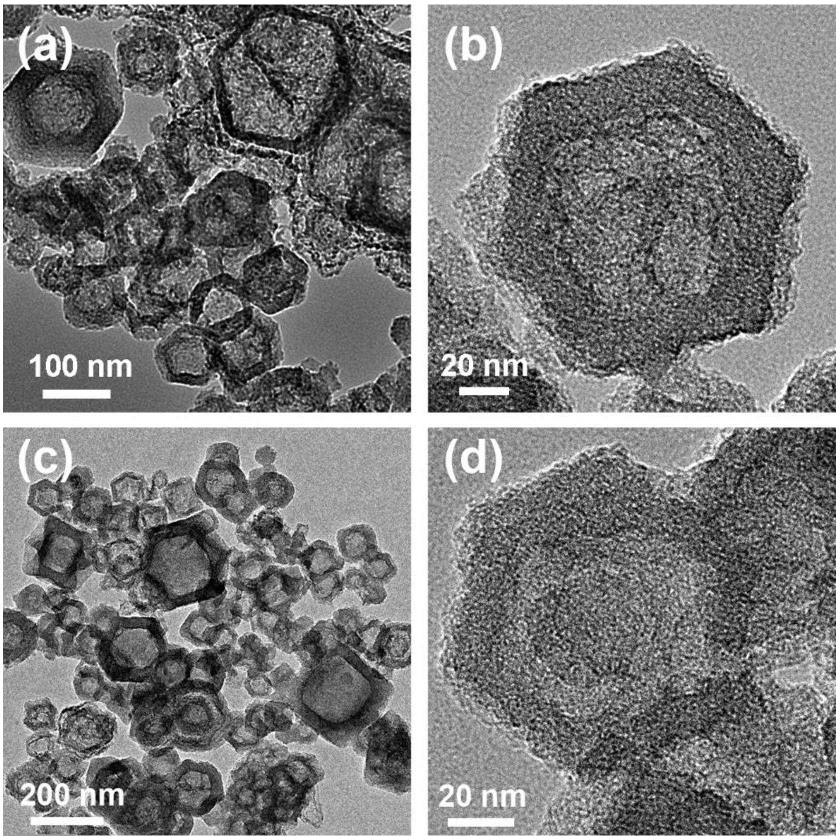

Figure S18. TEM images of (a, b) NC and (c, d) 0.79-ePt@NC.

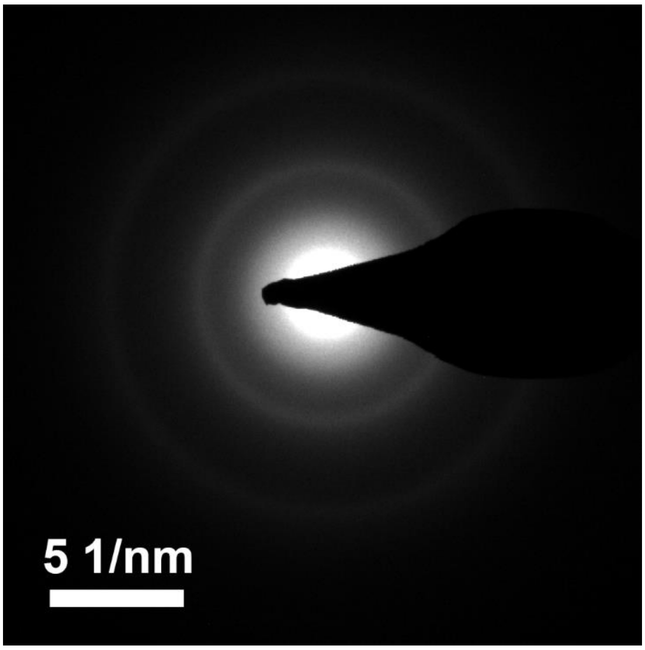

Figure S19. SAED image of 0.79-ePt@NC.

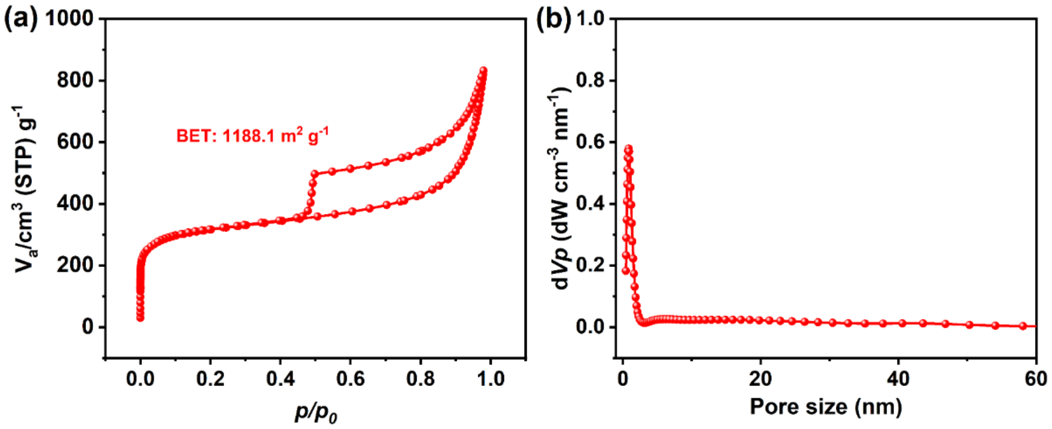

Figure S20. N_2 adsorption-desorption isotherms of 0.79-ePt@NC and pore size distribution.

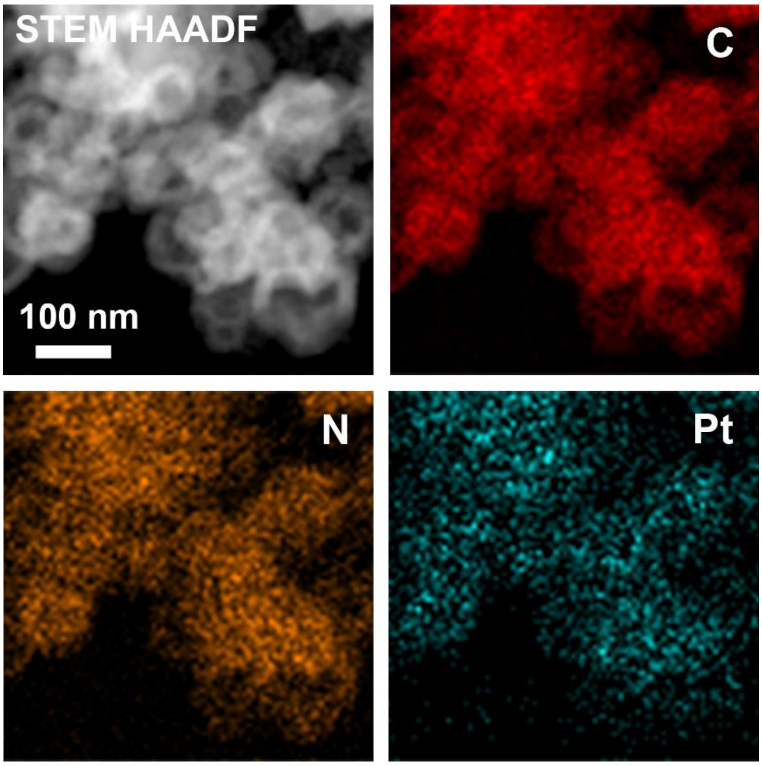

Figure S21. Low magnification HAADF-STEM and corresponding EDS mapping of 0.79-ePt@NC.

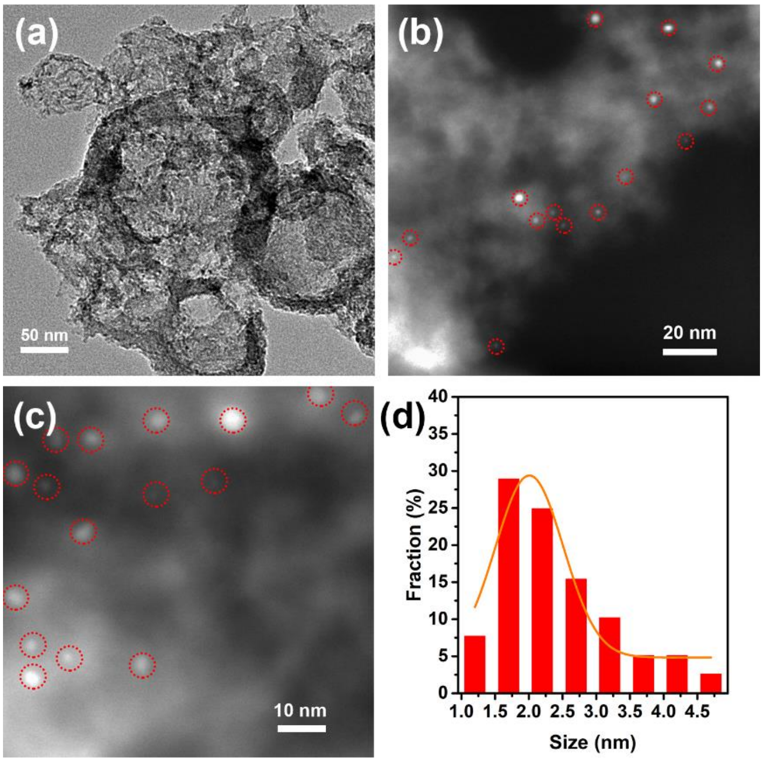

Figure S22. (a) TEM and (b, c) HAADF-STEM images of 1.83-ePt@NC. (d) Size distribution of Pt clusters in 1.83-ePt@NC.

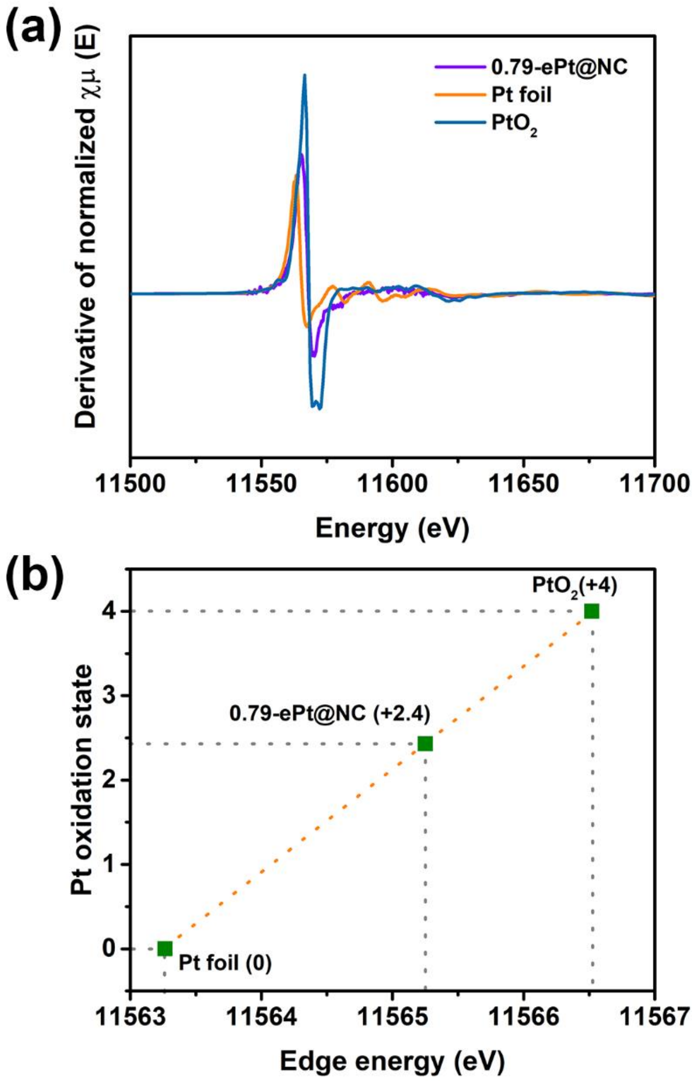

Figure S23. (a) First derivatives of Pt K-edge XANES regions of Pt foil, PtO_2, and 0.79-ePt@NC. (b) The fitted average oxidation states of Pt from XANES spectra.

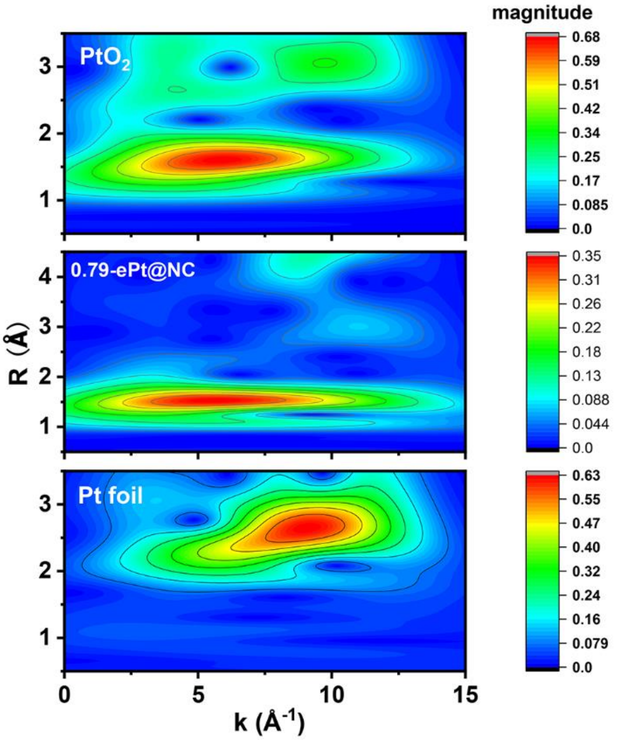

Figure S24. The wavelet transform of the 0.79-ePt@NC, Pt foil and PtO_2.

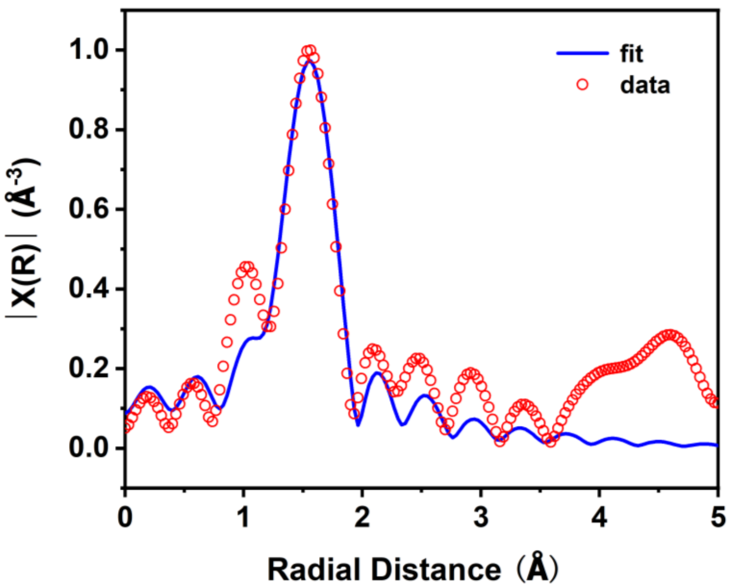

Figure S25. Experimental and fitting EXAFS curves of 0.79-ePt@NC in R space.

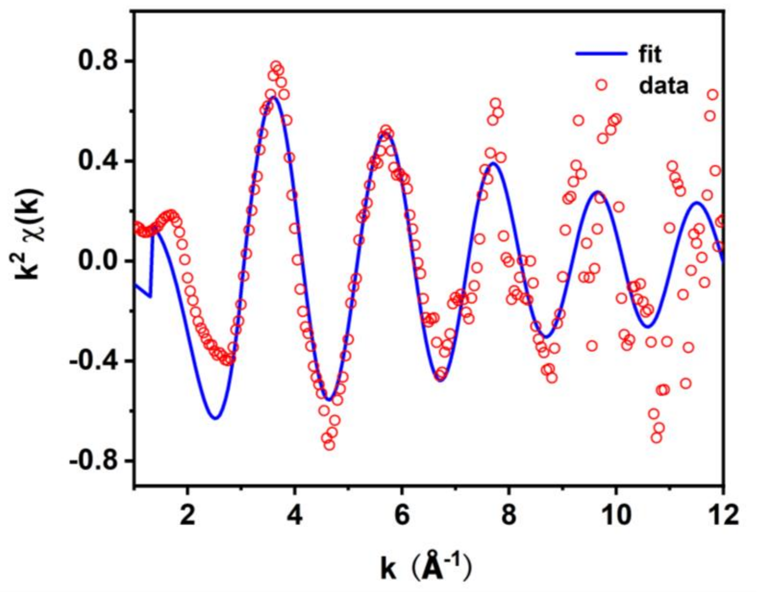

Figure S26. EXAFS fitting curve in k-space of 0.79-ePt@NC.

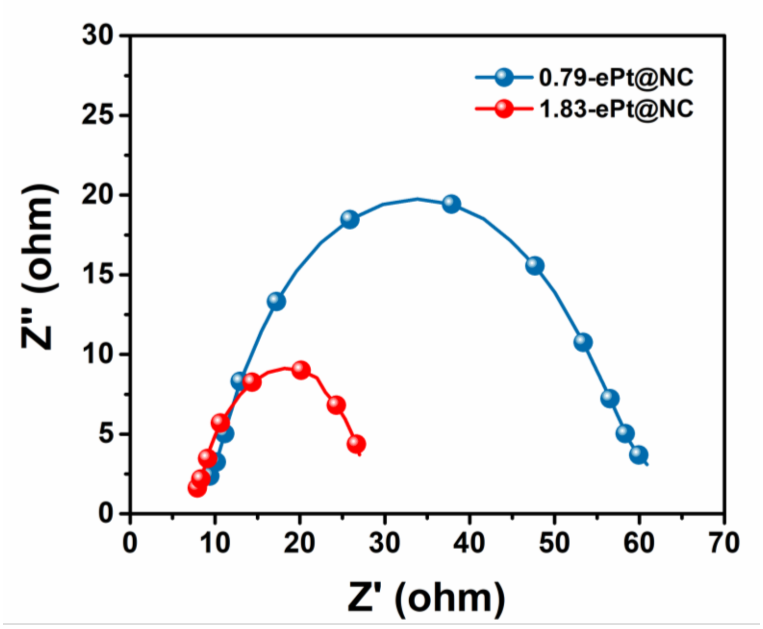

Figure S27. EIS of 0.79-ePt@NC and 1.83-ePt@NC.

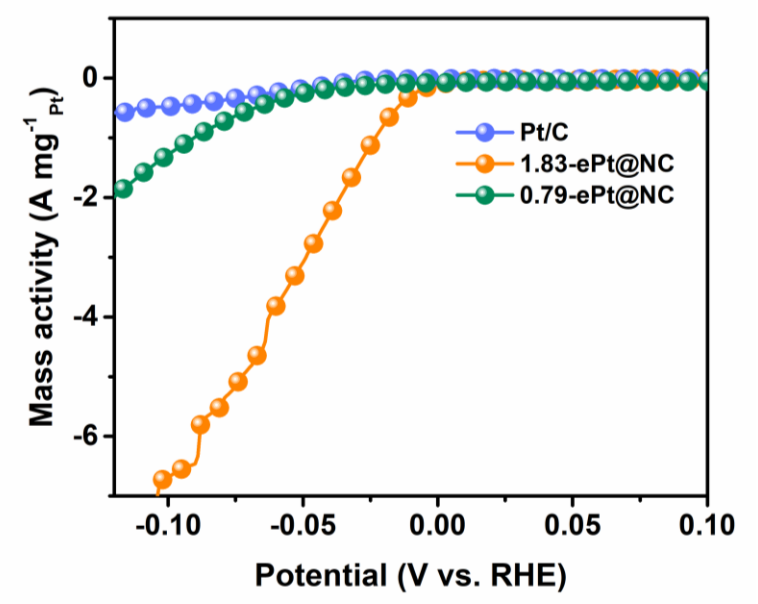

Figure S28. Pt mass loading normalized HER polarization curves of Pt/C, 1.83-ePt@NC, and 0.79-ePt@NC.

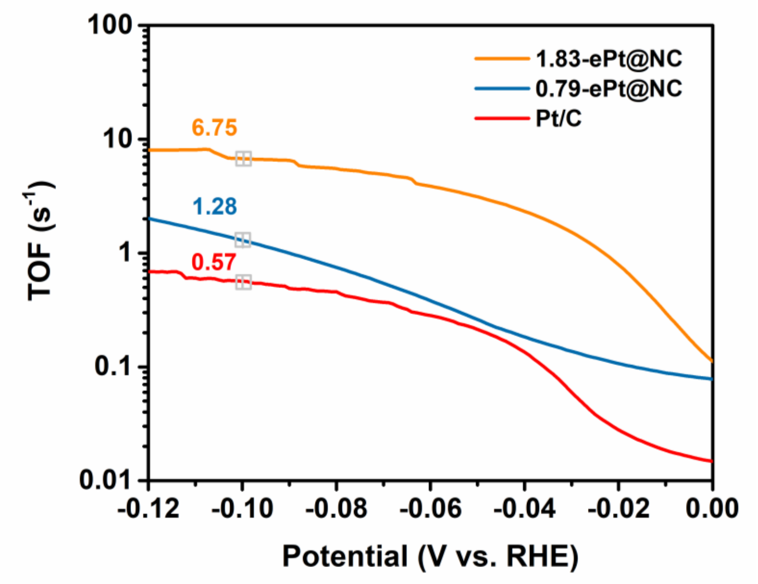

Figure S29. The TOF curves on 0.79-ePt@NC, 1.83-ePt@NC and commercial Pt/C.

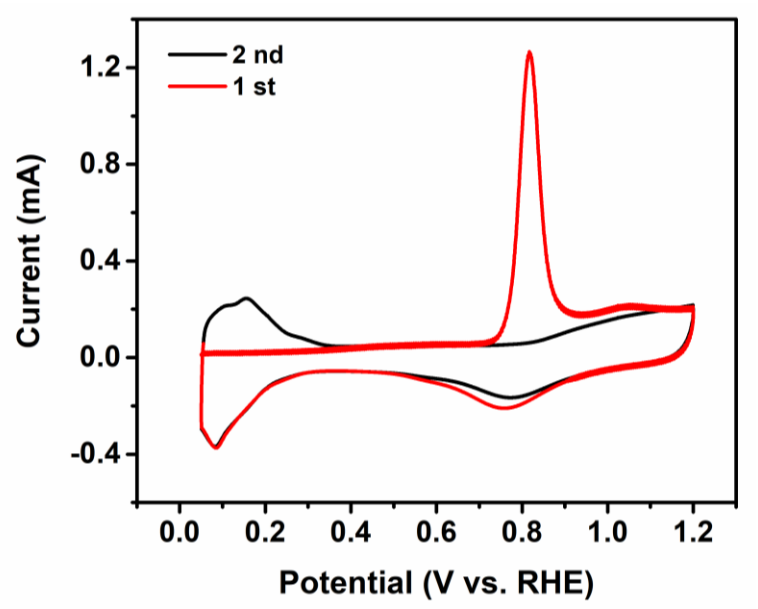

Figure S30. CO stripping measurements of commercial 20 wt% Pt/C catalyst.

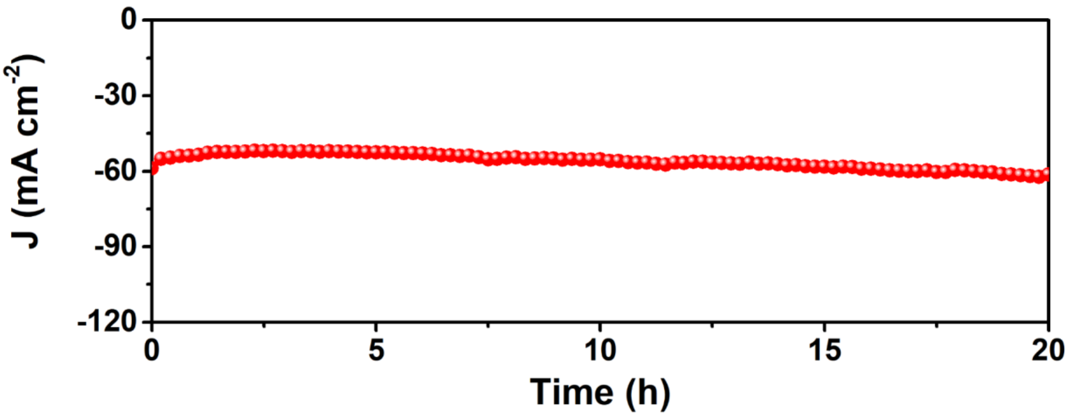

Figure S31. Long-term stability of 0.79-Pt@NC.

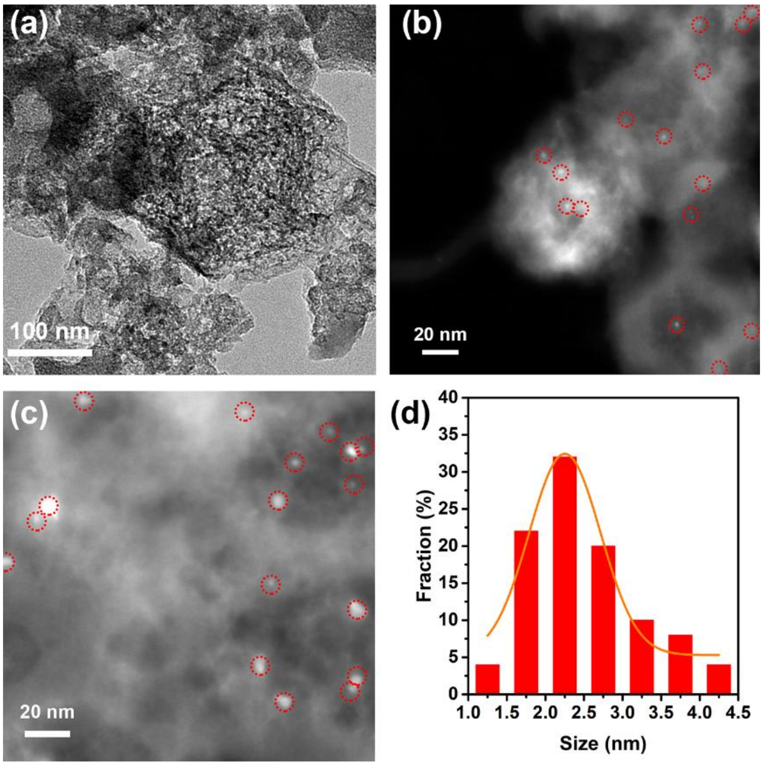

Figure S32. (a) TEM and (b, c) HAADF-STEM images of 1.83-Pt@NC after stability testing. (d) Size distribution of Pt clusters in 1.83-ePt@NC after stability testing.

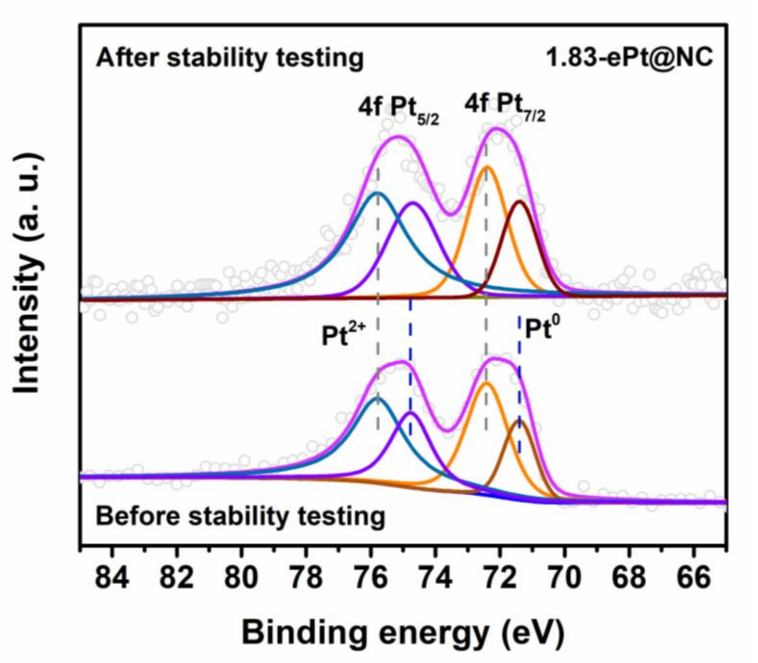

Figure S33. Comparison of high-resolution Pt 4f XPS spectra of 1.83-ePt@NC before and after electrocatalytic testing in 0.5 M H_2SO_4.

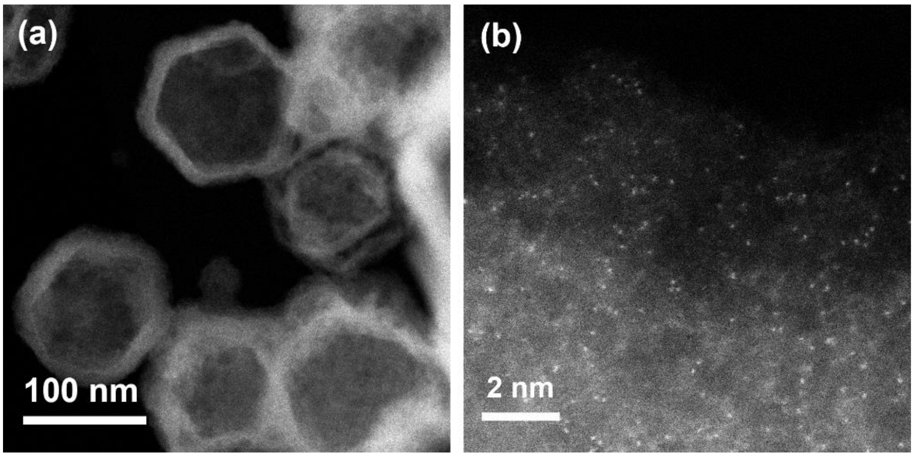

Figure S34. AC HAADF-STEM images of 0.79-ePt@NC after stability testing.

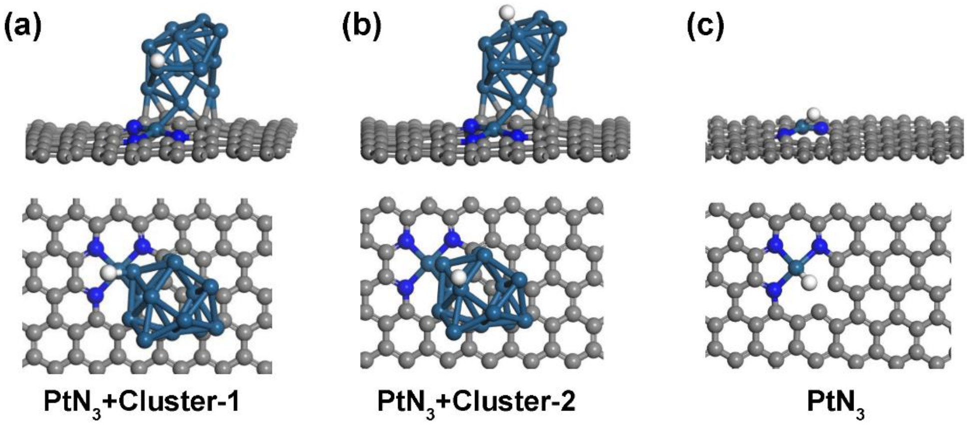

Figure S35. The top-view and side-view of the H-adsorption configurations of (a, b) Pt cluster/PtN_3 and (c) PtN_3.

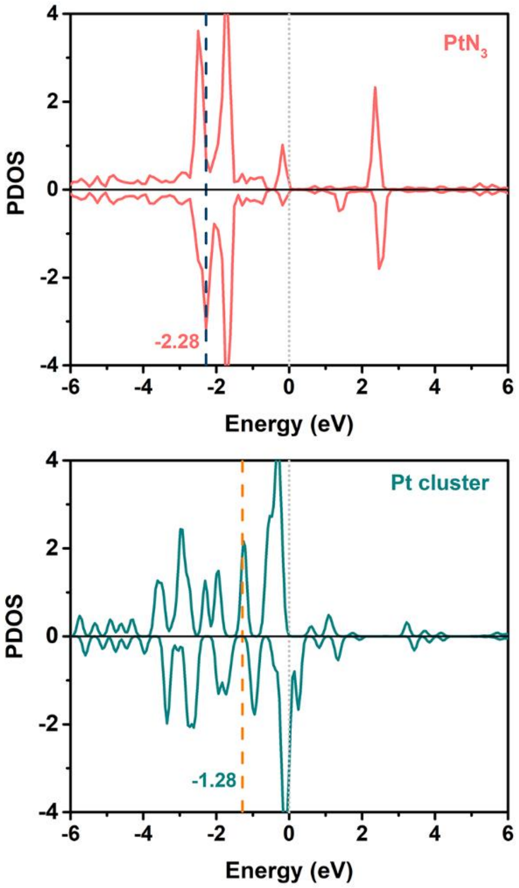

Figure S36. The PDOS plots of Pt cluster and PtN_3.

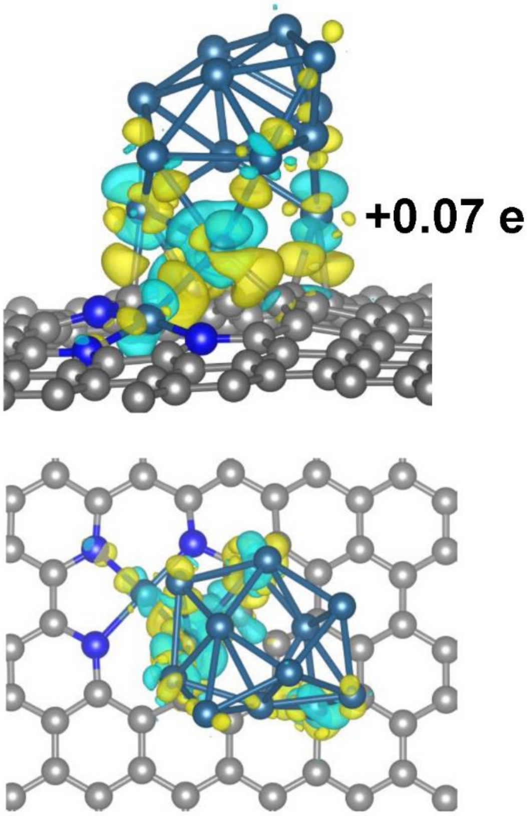

Figure S37. Charge density difference of Pt cluster/PtN_3.

Table S1. Fitting parameters for Pt L-edge EXAFS for the eZIF-Pt.

**Sample**	**Paths**	**CN**	**R**	**σ^2**
eZIF-Pt	Pt-N	1.98	2.66	0.004

S_0^2 was obtained from Pt foil and fixed as 0.72. ΔE_0 returned a value of 8.2026 ± 0.5557 eV. Data ranges $3.000 \leq k \leq 12.000$ Å^{-1}, $1.25 \leq R \leq 2.20$ Å. The number of variable parameters is 2, out of a total of 11.4375 independent data points, R factor for this fit is 0.69%. The Debye-Waller factors are obtained based on the *guessing* parameters and constrained as 0.004 for Pt-N. The delta *R* is based on *guessing* parameters and fixed as -0.13 for Pt-N.

Table S2. The amount of guest metal in ZIF-8.

sample	element	Metal concentration in eZIF-M (wt%)
eZIF-Ir	Ir	0.35
eZIF-Pd	Pd	0.46
eZIF-Ru	Ru	0.20
eZIF-Mo	Mo	0.11
eZIF-W	W	0.29
eZIF-Au	Au	0.86
eZIF-Ph	Rh	0.32
eZIF-Pt	Pt	0.38
0.73-ePt@NC	Pt	0.79
1.83-ePt@NC	Pt	1.83

Table S3. Fitting parameters for Pt L-edge EXAFS for the 0.79-ePt@NC.

Sample	Paths	CN[a]	R(Å)[b]	σ^2(Å^2)[c]	ΔE_0(eV)[d]	S_0^2	R factor
**0.79-ePt@NC**	Pt-N	3.0±0.2	1.98	0.001	6.9±0.7	0.8229	0.010

S_0^2 was obtained from Pt foil and fixed as 0.8229. [a]*CN*, coordination number; [b]*R*, the distance to the neighboring atom; [c]σ^2, the Mean Square Relative Displacement (MSRD); [d]ΔE_0, inner potential correction; *R* factor indicates the goodness of the fit. S_0^2 was fixed to 0.8229, according to the experimental EXAFS fit of the sample foil by fixing *CN* as the known crystallographic value. This value was fixed during EXAFS fitting, based on the known structure of Pt foil. Data ranges $3.0 \leq k \leq 11.0$ Å^{-1}, $1.0 \leq R \leq 2.0$ Å. The Debye-Waller factors and ΔRs are based on the *guessing* parameters and constrained for Pt-N.

Table S4. Comparison of HER activity for 1.83-ePt@NC in acidic solution with previously reported catalysts.

Ctalysts	η_{10} (mV)	Tafel slope (mV dec^{-1})	Ref
**$ALD_{50}Pt/NGNs$**	~38	29	[13]
**Pt-SA/NP-NC**	29.8	40.9	[14]
**$Mo_2TiC_2TxPtSA$**	30	30	[15]
**$Pt/npCo_{0.85}Se$**	55	35	[16]
**Pt_1/OLC**	38	35	[17]
**Pt-GT-1**	18	24	[18]
**PtW_6O_{24}/C**	22	30	[19]
**$F-SnO_2@Pt$**	42	34	[20]
**EG-Pt/CoP**	21	41	[21]
**$Pt@C_2N$**	52	50	[22]
**Pt QDs@Mxene**	33.3	29	[23]
**Pt SA/m-WO_3**	38	45	[24]
**$1Pt/VS_2/CP$**	77	40.13	[25]
**Pt-SAs/WS_2**	32	28	[26]
**Pt_5/HMCS-5.08%**	20.7	28.3	[27]
**CTAs@Pt@NCBs**	27.42	37.5	[28]
**CDs/Pt-PANI**	30	41.7	[29]
**Pt-GDY-2**	66	46.6	[30]
**Pt/MVF**	27	21	[31]
**1.83-ePt@NC**	20	28	**This work**

References

[1] B. Ravel, M. Newville, *J. Synchrotron Rad.* **2005**, 12, 537-541.
[2] H. Funke, A.C. Scheinost, M. Chukalina, *Phys. Rev. B* **2005**, 71, 094110.
[3] H. Funke, M. Chukalina, A.C. Scheinost, *J. Synchrotron Radiat.* **2007**, 14, 426-432.
[4] J.P. Perdew, K. Burke, M. Ernzerhof, *Phys. Rev. Lett.* **1997**, 78, 3865-3868.
[5] G. Kresse, J. Hafner, *Phys. Rev. B* **1993**, 47, 558-561.
[6] G. Kresse, J. Hafner, *Phys. Rev. B* **1994**, 49, 14251-14269.
[7] G. Kresse, J. Furthmüller, *Comp. Mater. Sci.* **1996**, 6, 15-50.
[8] G. Kresse, J. Furthmüller, *Phys. Rev. B* **1996**, 54, 11169.
[9] D.J. Chadi, *Phy. Rev. B* **1977**, 16, 1746-1747.
[10] J.K. Nørskov, J. Rossmeisl, A. Logadottir, L. Lindqvist, *J. Phys. Chem. B* **2004**, 108, 17886-17892.
[11] M. Jiao, W. Song, K. Li, Y. Wang, Z. Wu, *J. Phys. Chem. C* **2016**, 120, 8804-8812.
[12] Q. Yang, H. Liu, P. Yuan, Y. Jia, L. Zhuang, H. Zhang, X. Yan, G. Liu, Y. Zhao, J. Liu, S. Wei, L. Song, Q. Wu, B. Ge, L. Zhang, K. Wang, X. Wang, C.R. Chang, X. Yao, *J. Am. Chem. Soc.* **2022**, 144, 2171-2178.
[13] N. Cheng, S. Stambula, D. Wang, M.N. Banis, J. Liu, A. Riese, B. Xiao, R. Li, T.K. Sham, L.M. Liu, G.A. Botton, X. Sun, *Nat. Commun.* **2016**, 7, 13638.
[14] J. Liu, J. Liao, K. Huang, J. Dong, G. He, Z. Gong, H. Fei, *Adv. Mater.* **2023**, 35, 2211398.
[15] J. Zhang, Y. Zhao, X. Guo, C. Chen, C.-L. Dong, R.-S. Liu, C.-P. Han, Y. Li, Y. Gogotsi, G. Wang, *Nat. Catal.* **2018**, 1, 985-992.
[16] K. Jiang, B. Liu, M. Luo, S. Ning, M. Peng, Y. Zhao, Y.R. Lu, T.S. Chan, F.M.F. de Groot, Y. Tan, *Nat. Commun.* **2019**, 10, 1743.
[17] D. Liu, X. Li, S. Chen, H. Yan, C. Wang, C. Wu, Y.A. Haleem, S. Duan, J. Lu, B. Ge, P.M. Ajayan, Y. Luo, J. Jiang, L. Song, *Nat. Energy* **2019**, 4, 512-518.
[18] J.N. Tiwari, S. Sultan, C.W. Myung, T. Yoon, N. Li, M. Ha, A.M. Harzandi, H.J. Park, D.Y. Kim, S.S. Chandrasekaran, W.G. Lee, V. Vij, H. Kang, T.J. Shin, H.S. Shin, G. Lee, Z. Lee, K.S. Kim, *Nat. Energy* **2018**, 3, 773-782.
[19] F.Y. Yu, Z.L. Lang, L.Y. Yin, K. Feng, Y.J. Xia, H.Q. Tan, H.T. Zhu, J. Zhong, Z.H. Kang, Y.G. Li, *Nat. Commun.* **2020**, 11, 490.
[20] T. Kim, S.B. Roy, S. Moon, S. Yoo, H. Choi, V.G. Parale, Y. Kim, J. Lee, S.C. Jun, K. Kang, S.H. Chun, K. Kanamori, H.H. Park, *ACS Nano* **2022**, 16, 1625-1638.
[21] J. Li, H.-X. Liu, W. Gou, M. Zhang, Z. Xia, S. Zhang, C.-R. Chang, Y. Ma, Y. Qu, *Energ. Environ. Sci.* **2019**, 12, 2298-2304.
[22] C. Li, L. Zhang, Y. Zhang, Y. Zhou, J. Sun, X. Ouyang, X. Wang, J. Zhu, Y. Fu, *Chem. Eng. J.* **2022**, 428, 131085.
[23] S.Y. Pang, W.F. Io, J. Hao, *Adv. Sci.* **2021**, 8, 2102207.
[24] J. Park, S. Lee, H.E. Kim, A. Cho, S. Kim, Y. Ye, J.W. Han, H. Lee, J.H. Jang, J. Lee, *Angew. Chem. Int. Ed.* **2019**, 58, 16038-16042.
[25] J. Zhu, L. Cai, X. Yin, Z. Wang, L. Zhang, H. Ma, Y. Ke, Y. Du, S. Xi, A.T.S. Wee, Y. Chai, W. Zhang, *ACS Nano* **2020**, 14, 5600-5608.
[26] Y. Shi, Z.R. Ma, Y.Y. Xiao, Y.C. Yin, W.M. Huang, Z.C. Huang, Y.Z. Zheng, F.Y. Mu, R. Huang, G.Y. Shi, Y.Y. Sun, X.H. Xia, W. Chen, *Nat. Commun.* **2021**, 12, 3021.
[27] X.K. Wan, H.B. Wu, B.Y. Guan, D. Luan, X.W.D. Lou, *Adv. Mater.* **2020**, 32, 1901349.
[28] X. Chen, X. An, L. Tang, T. Chen, G. Zhang, *Chem. Eng. J.* **2022**, 429, 132259.
[29] Q. Dang, Y. Sun, X. Wang, W. Zhu, Y. Chen, F. Liao, H. Huang, M. Shao, *Appl. Catal. B-Environ.* **2019**, 257, 117905.
[30] X.P. Yin, H.J. Wang, S.F. Tang, X.L. Lu, M. Shu, R. Si, T.B. Lu, *Angew. Chem. Int. Ed.* **2018**, 57, 9382-9386.

[31] Q. Liang, W. Li, L. Xie, Y. He, B. Qiu, H. Zeng, S. Zhou, J. Zeng, T. Liu, M. Yan, K. Liang, O. Terasaki, L. Jiang, B. Kong, *Nano Lett.* **2022**, 22, 2889-2897.
